# Induction of Tet3-dependent Epigenetic Remodeling by Low-dose Hydralazine Attenuates Progression of Chronic Kidney Disease

**DOI:** 10.1016/j.ebiom.2014.11.005

**Published:** 2014-11-08

**Authors:** Björn Tampe, Desiree Tampe, Elisabeth M. Zeisberg, Gerhard A. Müller, Wibke Bechtel-Walz, Michael Koziolek, Raghu Kalluri, Michael Zeisberg

**Affiliations:** a Department of Nephrology and Rheumatology, Göttingen University Medical Center, Georg August University, Robert Koch Street 40, Göttingen, Germany; b Division of Matrix Biology, Department of Medicine, Beth Israel Deaconess Medical Center and Harvard Medical School, 330 Brookline Ave, Boston, MA, USA; c Department of Cardiology and Pneumology, Göttingen University Medical Center, Georg August University, Robert Koch Street 40, Göttingen, Germany; d German Center for Cardiovascular Research (DZHK), Robert Koch Street 40, Göttingen, Germany; e Renal Division, University Hospital Freiburg, Hugstetter Street 55, Freiburg, Germany; f Department of Cancer Biology and the Metastasis Research Center, University of Texas MD Anderson Cancer Center, 1515 Holcombe Blvd, Houston, TX, USA

**Keywords:** Epigenetics, DNA methylation, Fibrosis, Ten-Eleven Translocation (TET), De-methylation, RASAL1

## Abstract

Progression of chronic kidney disease remains a principal problem in clinical nephrology and there is a pressing need for novel therapeutics and biomarkers. Aberrant promoter CpG island methylation and subsequent transcriptional silencing of specific genes have emerged as contributors to progression of chronic kidney disease. Here, we report that transcriptional silencing of the Ras-GTP suppressor RASAL1 contributes causally to progression of kidney fibrosis and we identified that circulating methylated *RASAL1* promoter DNA fragments in peripheral blood correspond with levels of intrarenal levels of *RASAL1* promoter methylation and degree of fibrosis in kidney biopsies, enabling non-invasive longitudinal analysis of intrarenal CpG island methylation.

Retrospective analysis of patients with hypertensive nephrosclerosis revealed that circulating methylated *RASAL1* promoter DNA fragments in peripheral blood decrease with Dihydralazine treatment in patients with hypertensive nephrosclerosis, and provided evidence that low-dose Dihydralazine delays decline of excretory kidney function, whereas Dihydralazine at standard doses had no protective effect. We demonstrate that the protective effect of Dihydralazine is due to induction of endogenous Tet3/Tdg-mediated DNA-de-methylation activity reversing aberrant promoter CpG island methylation, while HIF1α induction at standard doses counterbalances its protective activity. We conclude that *RASAL1* promoter methylation is a therapeutic target and a biomarker of renal fibrosis. Our study suggests that therapeutic use of low-dose Dihydralazine in patients with chronic kidney disease and fibrosis deserves further consideration.

## Highlights

• Aberrant RASAL1 promoter methylation contributes causally to progression of kidney fibrosis.
• Degree of intrarenal RASAL1 methylation is reflected by levels of circulating methylated RASAL1 promoter fragments.
• Low-dose Hydralazine induces endogenous Tet3-dependent de-methylation and inhibits progression of kidney fibrosis.

## 1 Introduction

Progression of chronic kidney disease (CKD) remains an unsolved problem in nephrology as effective drugs to slow down or even reverse CKD progression are not yet available for clinical use (Science JMECfCDaC, 2012). Because the common pathological alteration in virtually every progressive CKD is renal fibrosis, several anti-fibrotic agents are among leading candidates for CKD and have finally entered clinical testing (Tampe and Zeisberg, 2014a). However, it may still take years until new treatment regimens are approved and established for clinical practice. Current management of CKD mainly aims at treating underlying conditions and cardiovascular risk factors (Science JMECfCDaC, 2012). As CKD is commonly associated with hypertension, most prevalent pharmacological interventions include angiotensin-converting enzyme inhibitors (ACEIs), angiotensin-II receptor blockers (ARBs), beta blockers, diuretics, statins and calcium channel blockers (Science JMECfCDaC, 2012, Rayner et al., 2014). Of these, only ACEIs and ARBs have demonstrated the ability to slow progression of CKD, albeit only in patients with marked proteinuria, these classes of drugs are currently considered first-line treatments (James et al., 2014). Due to the imminent clinical need for effective anti-fibrotic therapies, identification of additional, already approved drugs with reno-protective potential would be highly desirable.

In this regard, aberrant methylation of genomic DNA has emerged as a novel therapeutic target. DNA methylation in general refers to methylation of cytosine bases that are clustered in so-called CpG-islands. Methylation of CpG islands within promoter regions effectively silences expression of affected genes. While methylation of promoter CpG islands is a physiological mechanism to determine cell lineage and differentiation, aberrant promoter methylation (typically referred to as hypermethylation) and subsequent transcriptional silencing of affected genes are potent contributors to carcinogenesis. Recent studies demonstrated that aberrant promoter CpG island methylation also plays a role in renal fibrosis (and in fibrosis involving other organs as well) (Bechtel et al., 2010, Tampe et al., 2014). The importance of aberrant promoter CpG island methylation is highlighted by the effectiveness of 5′-Azacytidine, the first de-methylating agent which was approved for clinical use for patients with high-risk myelodysplastic syndrome (MDS) and acute myeloid leukemia (Zeisberg and Zeisberg, 2013). With regard to chronic kidney disease, administration of 5′-Azacytidine ameliorated fibrosis in several models of experimental chronic kidney injury (Bechtel et al., 2010). 5′-Azacytidine is a broad de-methylating agent which – as a cytidine analog – inhibits DNA methyltransferase 1 (DNMT1) and is incorporated into the DNA of dividing cells prompting DNA repair and replacement of methylated CpGs with naked cytosine (Foulks et al., 2012, Tampe and Zeisberg, 2014b, Tampe and Zeisberg, 2012). Due to DNA-incorporation, 5′-Azacytidine and its derivate 5′-Aza-2′-Deoxycytidine have considerable cytotoxicity in addition to their unspecific de-methylating activity. Hence, more specific normalization of aberrant hypermethylation without incorporation of potentially toxic nucleotide analogs would be much more attractive.

In this regard, Hydralazine (syn. 1-Hydrazinylphthalazine) was primarily synthesized by Ciba as an anti-hypertensive compound and has long been known to possess de-methylating activity (Arce et al., 2006, Druey, 1949). In clinical studies, Hydralazine and its derivate Dihydralazine were effective in de-methylating and re-activating expression of aberrantly silenced genes in cancer patients and a recent phase III clinical study demonstrated delayed cancer progression in patients receiving Hydralazine (Coronel et al., 2011). After its introduction to the clinic in 1952 as an anti-hypertensive drug, Hydralazine became part of the standard triple-regimen (Hydrochlorothiazide, Propranolol and Hydralazine) (Heagerty et al., 1982). With the emergence of ACEIs and ARBs as anti-hypertensive drugs with cardio-protective activity, Hydralazine today is mainly used as a third-line drug in patients with therapy-resistant hypertension (Leonetti et al., 1990). Due to it being highly safe, it is still a first-line therapy for hypertension during pregnancy (James et al., 2014). To our knowledge, its effectiveness to decrease the prevalence of ESRD has not yet been assessed. The aim of this study was to elucidate the potential of Hydralazine to correct aberrant DNA methylation in fibrotic kidneys and subsequently inhibit renal fibrogenesis.

## 2 Materials and Methods

### 2.1 Unilateral Ureteral Obstruction (UUO)

Eight to twelve week old *CD57BL/6* mice were anesthetized with isoflurane inhalation, analgesia was performed by subcutaneous Buprenorphine injection. The ureter was separated from the surrounding tissues and two ligatures were placed about 5 mm apart in the upper two-thirds of the ureter of the left kidney to obtain reliable obstruction. On the same day after surgery and recovery, mice were treated with either vehicle buffer PBS, 5′-Azacytidine (10 mg/kg/day, Sigma, St. Louis, USA), low-dose, or high-dose Hydralazine (5 mg/kg/day or 50 mg/kg/day, respectively, Sigma, St. Louis, USA) given intraperitoneally every other day. Mice were sacrificed at indicated time points after ureter ligation (Tampe et al., 2014).

### 2.2 Folic Acid-induced Nephropathy (FA)

Kidney injury was induced with a single intraperitoneal injection of folic acid (250 mg/kg body weight in PBS) in *CD1* mice. Injections with vehicle buffer PBS, 5′-Azacytidine (10 mg/kg/day, Sigma, St. Louis, USA), and Hydralazine (5 mg/kg/day or 50 mg/kg/day, respectively, Sigma, St. Louis, USA) were done intraperitoneally every other day on day three after induction of folic acid until day 28. Mice were sacrificed 96 and 147 days after induction.

### 2.3 *rtTAhCMV;hRASAL1-pTreTight* Transgenic Mice

From three days before unilateral ureteral obstruction (UUO) on, *wildtype* and *rtTAhCMV;hRASAL1-pTreTight* transgenic mice received either vehicle buffer or doxycycline (DOX, 2 mg/ml, Clontech, Mountain View, USA) within drinking water and renewed every two days. Mice were sacrificed at indicated time points after ureteral ligation.

### 2.4 Histology

Paraffin-embedded kidneys were sectioned at 3 μm and Masson's Trichrome Stain (MTS) was performed at the BIDMC Histopathology Core and the University Medical Center Göttingen. We assessed the fibrotic area using cellSens (Olympus, Tokyo, Japan) software, as previously described (Tampe et al., 2014, Sugimoto et al., 2012).

### 2.5 Immunofluorescence

For immunofluorescent staining, primary antibodies against α-smooth muscle actin (αSMA, Abcam, Cambridge, UK) and fibroblast-specific protein 1 (Fsp1, Sigma, St. Louis, USA), and Alexa Fluor 568 (Invitrogen, Carlsbad, USA) secondary antibodies were used. Nuclear staining was performed using 4′,6-diamidino-2-phenylindole (DAPI, Vector Labs, Burlingame, USA). Relative areas positive for αSMA and Fsp1 per visual fields were analyzed at original magnification × 40, representative confocal pictures are shown at original magnification × 63.

### 2.6 Serum Creatinine Measurement

Creatinine concentration was measured using the colorimetric assay Quantichrome DICT-500 (BioAssays, Hayward, USA) according the manufacturer's directions, as previously described (Sugimoto et al., 2012, LeBleu et al., 2009).

### 2.7 Cell Culture

Primary murine kidney fibroblasts were isolated as described in our previous publications with minor modifications (Bechtel et al., 2010, Tampe et al., 2014). Kidneys were harvested and cultured in DMEM (Gibco, Carlsbad, USA) supplemented with 2 mmol/l l-glutamine, 100 g/ml penicillin, 100 g/ml streptomycin and 20% heat-inactivated fetal bovine serum (FBS, Cellgro, Manassas, USA) at 37 °C in 5% CO_2_. Primary kidney fibroblasts emerged within few days as adherent cells on the monolayer and were maintained in DMEM (Gibco, Carlsbad, USA) supplemented with 2 mmol/l l-glutamine, 100 g/ml penicillin, 100 g/ml streptomycin and 10% heat-inactivated fetal bovine serum (FBS, Cellgro, Manassas, USA) at 37 °C in 5% CO_2_. Fibroblasts of passages 2 to 4 were used. For RNA and DNA extraction, primary murine kidney fibroblasts were incubated with serum-free media containing 5′-Azacytidine (100 or 150 μg/ml in PBS, Sigma, St. Louis, USA), Hydralazine (10 or 50 μg/ml in PBS, Sigma, St. Louis, USA) or doxycycline (DOX, 1 μg/ml, Clontech, Mountain View, USA). Cells were harvested for further analysis one and two days after incubation.

### 2.8 In Vitro Transfection

Cells were seeded in culture flasks in antibiotic-free DMEM (Gibco, Carlsbad, USA) supplemented with 10% heat-inactivated fetal bovine serum (FBS, Cellgro, Manassas, USA). For knockdown experiments, 600 pmol siRNA or scrambled siRNA (Santa Cruz Biotechnology, Santa Cruz, USA) was transfected using Lipofectamine 2000 reagent (Invitrogen, Carlsbad, USA). After 12 h of incubation, transfection medium was replaced, cells were seeded in six-well culture plates at a concentration of 60,000 per well and incubated with serum-free media containing 5′-Azacytidine (100 or 150 μg/ml in PBS, Sigma, St. Louis, USA) or Hydralazine (10 or 50 μg/ml in PBS, Sigma, St. Louis, USA). Cells were harvested for further analysis after 24 and 48 h of incubation.

### 2.9 RNA Isolation

Cells were dissolved in TRIzol (Invitrogen, Carlsbad, USA), tissue was shredded using TissueLyser LT (Qiagen, Hilden, Germany). RNA was isolated using PureLink RNA Mini Kit (Ambion, Carlsbad, USA) according to the manufacturer's protocol.

### 2.10 Quantitative Real-time PCR Quantification (qRT-PCR)

For SYBR-based real-time PCR, cDNA synthesis was performed by using DNase I digestion and SuperScript II Reverse Transcriptase (Invitrogen, Carlsbad, USA) according to the manufacturer's protocol. 1 μl of reverse-transcribed cDNA was added to the reaction mixture containing the primer pair (200 nmol/l each) and diluted 2 × Fast SYBR Green Master Mix (Applied Biosystems, Carlsbad, USA) in a final volume of 20 μl for each PCR reaction. The real-time PCR reactions were performed in a 96-well reaction plate using the StepOne Real-Time System (Applied Biosystems, Carlsbad, USA) and were done in triplicates. An initiation step at 95 °C for 20 s was followed by 40 cycles at 95 °C for 3 s and 60 °C for 30 s, with 1 cycle of dissociation at 95 °C for 15 s, 60 °C for 60 s, and 95 °C for 15 s. The intercalation of SYBR Green dye and its fluorescent signal is directly proportional to the amount of amplified DNA and was transformed into the cycle threshold (Ct). For normalization, the Ct values of the housekeeping gene were subtracted from the Ct values of the gene of interest to generate the dCt values. The relative expression levels were calculated using the equation 2^− ddCt^. Oligonucleotide sequences are shown in Supplementary Table A.

### 2.11 DNA Isolation

Tissue and cells were dissolved in TRIzol (Invitrogen, Carlsbad, USA), tissue was shredded using TissueLyser LT (Qiagen, Hilden, Germany). DNA from tissue, cells and peripheral blood samples was isolated using DNeasy Blood & Tissue Kit (Qiagen, Hilden, Germany) according to the manufacturer's protocol.

### 2.12 Isolation of Methylated, Hydroxymethylated, Carboxylated DNA

Methylated DNA was isolated from 1.0 μg of sonicated DNA using Methylamp Methylated DNA Capture (MeDIP) Kit, hydroxymethylated DNA was isolated from 0.5 μg of sonicated DNA using EpiQuik Hydroxymethylated DNA Immunoprecipitation (hMeDIP) Kit (Epigentek, Farmingdale, USA). Carboxylated DNA was isolated using 1.0 μg of sonicated DNA for immunoprecipitation with 5-carboxyl-cytosine antibody (Active Motif, Carlsbad, USA). DNA was added to each antibody coated well and incubated for 120 min at room temperature on an orbital shaker. After releasing with proteinase K for 60 min at 65 °C, DNA was eluted from the column and adjusted to a final volume of 100 μl with nuclease-free water. For each sample, an input vial was performed using total sonicated DNA for further normalization.

### 2.13 SYBR-based Amplification of Methylated, Hydroxymethylated, Carboxylated Rasal1/RASAL1

For DNA amplification, 5 μl of eluted DNA was added to the reaction mixture containing the primer pair (300 nmol/l each), a ROX passive reference dye (Bio-Rad, Hercules, USA) and diluted 2 × SYBR green Supermix (Bio-Rad, Hercules, USA) in a final volume of 25 μl for each PCR reaction. The real-time PCR reactions were performed in a 96-well reaction plate using the Mx3000P QPCR System (Stratagene, Santa Clara, USA). PCR reaction was stopped when the fluorescent signal increased over the threshold and electrophoresis of PCR products was done on a Bioanalyzer 2100 (Agilent Technologies, Santa Clara, USA) according to the manufacturer's protocol. Electrophoresis results are shown as virtual gel images as described in our previous publications (Bechtel et al., 2010, Tampe et al., 2014). For quantification of intrarenal and circulating methylated *Rasal1/RASAL1* promoter DNA fragments, the Ct values of the input samples were subtracted from the Ct values of the MeDIP samples after enrichment to generate the dCt values used in the equation 2^− dCt^. Oligonucleotide sequences are shown in Supplementary Table B.

### 2.14 Methylation-specific PCR

Bisulfite conversion was performed using Cells-To-CpG Bisulfite Conversion Kit (Applied Biosystems, Carlsbad, USA) according to the manufacturer's protocol. Briefly, 2 μg genomic DNA was denaturated for 10 min at 50 °C, conversion of unmethylated cytosines to uracil was performed in 2 cycles at 65 °C for 30 min, followed by 95 °C for 90 s using a thermal cycler (Eppendorf, Hamburg, Germany). Methylation-specific PCR was performed using primers specifically targeting converted (unmethylated) or unconverted (methylated) CpG sites. Oligonucleotide sequences are shown in Supplementary Table C.

### 2.15 Western Blot Analyses

Tissue and cells were homogenized in NP40 lysis buffer (Invitrogen, Carlsbad, USA) supplemented with protease cocktail inhibitor (Roche, Basel, Switzerland). After sonication, proteins were resolved by Tris-acetate-SDS acrylamide gel electrophoresis and transferred onto PVDF membranes (Invitrogen, Carlsbad, USA) and blocked in 5% dry milk in TBS-T (TBS pH 7.6, 0.1% Tween-20). After incubation with respective primary antibodies against Dnmt1 (B-Bridge, Cupertino, USA), Tet3 (Genetex, San Antonio, USA), Tdg (Santa Cruz Biotechnology, Santa Cruz, USA), HIF1α (Invitrogen, Carlsbad, USA), β-actin (Sigma, St. Louis, USA) and Gapdh (HyTest, Turku, Finland), secondary HRP-conjugated antibodies were used (Dako, Glostrup, Denmark). Luminescence was detected on a ChemiDoc XRS system (Bio-Rad, Hercules, USA) using chemiluminescent substrate (Cell Signaling, Danvers, USA).

### 2.16 Kidney Biopsies and Human Peripheral Blood Samples

Detailed clinical patient data are presented in Supplementary Tables D–G.

### 2.17 Statistical Analysis

ANOVA was used for multiple comparisons of mouse samples to determine significance. Student's t-test analysis was used for the single-parameter comparisons. Statistical significance was defined as values of *p < 0.05. Prism 5 software (GraphPad, La Jolla, USA) was used for statistical analysis.

### 2.18 Ethical Research Conduct

All experimental animal studies have been carried out with the approval of the Institutional Animal Care and Use Committee of the Beth Israel Deaconess Medical Center or the University Medical Center Göttingen. The use of parts of kidney biopsies and peripheral blood samples for research purposes was approved by the Ethics Committee of the University Medical Center Göttingen, and written consent was obtained from all subjects before kidney biopsy.

## 3 Results

### 3.1 Hydralazine Ameliorates Experimental Renal Fibrosis in Mice

**Fig. 1:**
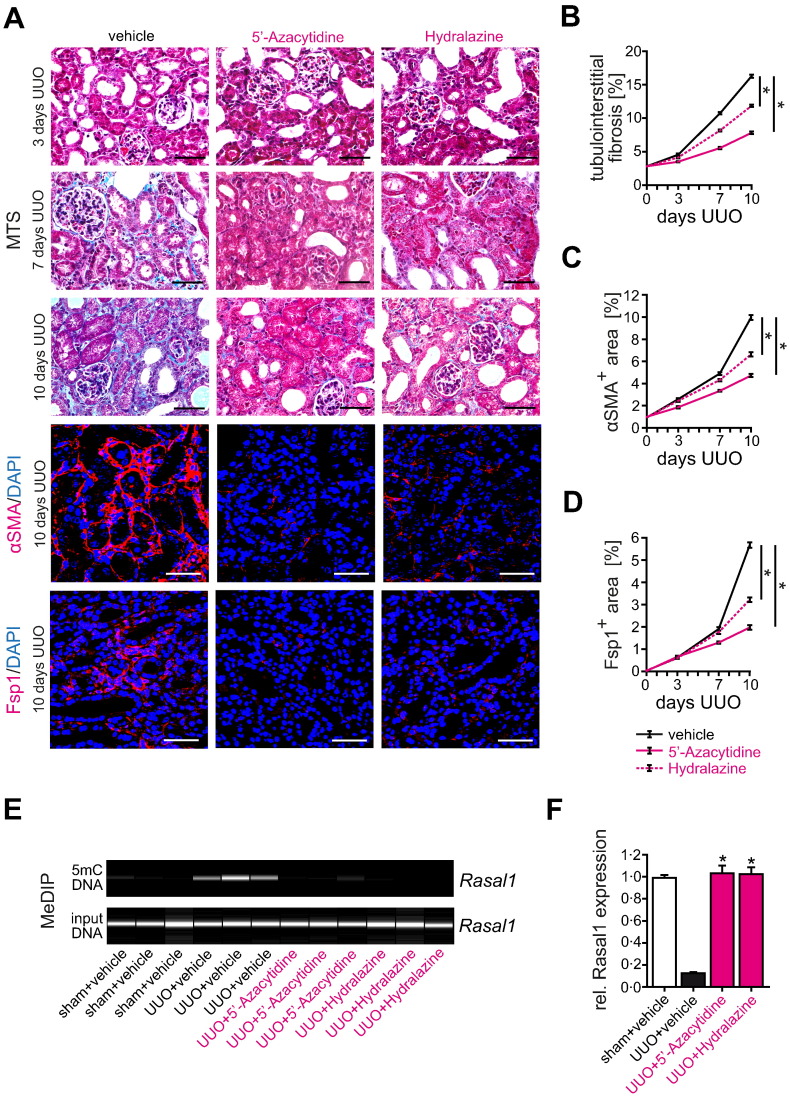
Hydralazine administration ameliorates experimental kidney fibrosis and fibroblast accumulation in the mouse model of unilateral ureteral obstruction (UUO). (A) Representative photomicrographs of Masson's trichrome-stained (MTS) kidneys or sections immunolabeled with primary antibodies against α-smooth muscle actin (αSMA) or fibroblast-specific protein 1 (Fsp1). The panels show fibrotic kidney sections from mice challenged with UUO at indicated time points after ureteral obstruction. As compared to control mice receiving vehicle buffer, treatment with de-methylating 5′-Azacytidine or Hydralazine attenuated renal fibrogenesis and fibroblast accumulation (scale bars: 50 μm). (B–D) The time course summarizes average mean values at the indicated time points of each group (n = 6 in each group, data are presented as means ± s.e.m. *p < 0.05, values of *p* were calculated respective to vehicle-treated UUO mice). (E) We performed methylated DNA immunoprecipitation (MeDIP) to assess the effect of anti-fibrotic 5′-Azacytidine and Hydralazine treatment (10 mg/kg/day or 5 mg/kg/day respectively) on *Rasal1* promoter methylation in mouse kidneys that were challenged with unilateral ureteral obstruction (UUO). In this assay, fragmented total kidney DNA (input DNA) was exposed to antibodies that specifically capture methylated DNA (5mC DNA). The captured DNA is eluted and analyzed by primers specific to *Rasal1* promoter, electrophoresis of PCR products was performed on a Bioanalyzer. The pictures display virtual gel images of *Rasal1* PCR products of captured (methylated, 5mC) DNA, the bottom pictures display *Rasal1* PCR products of input DNA (as controls for equal loading in immunoprecipitation). *Rasal1* was hypermethylated when kidneys had developed severe tubulointerstitial fibrosis ten days after ureteral obstruction (UUO + vehicle), whereas *Rasal1* hypermethylation was ameliorated in mice treated with de-methylating 5′-Azacytidine (UUO + 5′-Azacytidine) or Hydralazine (UUO + Hydralazine). (F) Rasal1 mRNA expression was analyzed by qRT-PCR in RNA isolated from total kidneys. For statistical analysis, UUO mice treated with vehicle buffer PBS, with de-methylating 5′-Azacytidine, or Hydralazine were compared to sham-operated controls arbitrarily set to one. Rasal1 expression was suppressed in kidneys of mice which had been challenged with UUO and which had received vehicle buffer PBS, but was restored in kidneys of mice treated with 5′-Azacytidine or Hydralazine (n = 4 in each group, experiments were done in triplicate, data are presented as means ± s.e.m. *p < 0.05, values of *p* were calculated respective to vehicle-treated UUO mice).

Based on encouraging studies demonstrating the de-methylating activity of Hydralazine with regard to cancer cells, we first aimed to determine if this de-methylating activity could be utilized to inhibit renal fibrogenesis, as was previously shown for the prototypical (yet more toxic) de-methylating drug 5′-Azacytidine (Bechtel et al., 2010). For this purpose, we opted to challenge *C57BL/6* mice with unilateral ureteral obstruction (UUO) and to administer Hydralazine intraperitoneally at a dose of 5 mg/kg/day, which has been established as an optimum regimen in murine cancer studies (Chavez-Blanco et al., 2006). We chose the UUO model because fibrosis in the obstructed kidney develops without hypertension, pre-empting possible misinterpretation due to possible anti-hypertensive activity of Hydralazine (Fern et al., 1999, Ma et al., 2003, Manucha et al., 2004, Sugiyama et al., 2005). To directly compare the effectiveness of Hydralazine to that of 5′-Azacytidine, we also included a cohort of mice which received 5′-Azacytidine (10 mg/kg/day intraperitoneally as established as an optimum dose in previous studies), and we sacrificed cohorts of mice at pre-determined time-points for tissue analysis (Bechtel et al., 2010). After 10 days of ureteral obstruction, kidneys of mice that had received either 5′-Azacytidine or Hydralazine displayed significantly less fibrosis, as compared to kidneys of control UUO-challenged mice (which had received vehicle buffer PBS) (Fig. 1A, B). Ameliorated fibrosis correlated with blunted accumulation of fibroblasts (determined by quantification of αSMA and Fsp1 immunolabeling) (Fig. 1A–D). To further validate this observation, we aimed to challenge mice of different genetic backgrounds with a disease model of distinct pathomechanism. For this purpose, we challenged *CD1* mice with a single injection of folic acid (FA, which results in severe renal fibrosis within 5 months) and administered 5′-Azacytidine or Hydralazine at 10 mg/kg/day and 5 mg/kg/day, respectively, from day 3 to day 21 after the folic acid injection. Rationale for using this approach was that we previously established this treatment schedule to be effective when 5′-Azacytidine was administered in this model (Bechtel et al., 2010). After 5 months, kidneys developed severe tubulointerstitial fibrosis, however, kidneys of mice which received 5′-Azacytidine or Hydralazine displayed significantly less fibrosis as compared to kidneys of control mice (which received vehicle buffer PBS) (Supplementary Fig. 1A, B). Ameliorated fibrosis in mice which received 5′-Azacytidine or Hydralazine correlated with blunted rise of serum creatinine levels (Supplementary Fig. 1C) and ameliorated fibroblast accumulation (Supplementary Fig. 2A–D). As compared to Hydralazine-treated mice, fibrosis and plasma creatinine levels were lower in 5′-Azacytidine-treated mice, albeit differences between the groups did not reach statistical significance (Supplementary Figs. 1A–C and 2A–D).

### 3.2 Reno-Protective Effect of Hydralazine and Rasal1 Promoter CpG Island De-methylation

We next aimed to elucidate if ameliorated fibrosis observed upon Hydralazine treatment was due to its de-methylating efficacy. For this purpose, we analyzed *Rasal1* promoter CpG island methylation in genomic DNA samples isolated from total kidney tissues. We focused on *Rasal1* methylation because we previously identified *RASAL1* (encoding for RASAL1, synonym Ras-GAP-like protein, and inhibitor of Ras-GTP) to be selectively methylated in fibrotic kidney biopsies utilizing a genome-wide methylation screen and we verified that *Rasal1* promoter CpG island methylation correlated with degree of fibrosis in murine models of renal fibrosis (including UUO and folic acid-induced nephropathy) as well as in renal biopsies (Bechtel et al., 2010, Tampe et al., 2014). Fibrosis upon challenge with UUO was associated with increased *Rasal1* CpG island promoter methylation (Fig. 1E), whereas ameliorated fibrosis upon Hydralazine administration correlated with reduced *Rasal1* methylation, similar to observations after administration of the prototypical de-methylating agent 5′-Azacytidine (Fig. 1E). A similar association of fibrosis, anti-fibrotic efficacy of 5′-Azacytidine or Hydralazine administration and degree of *Rasal1* CpG island promoter methylation was documented when kidneys of folic acid-challenged mice were analyzed (Supplementary Fig. 3A). Normalization of *Rasal1* CpG island promoter methylation upon treatment with 5′-Azacytidine or Hydralazine was associated with normalization of *Rasal1* mRNA expression in both mouse models used (Fig. 1F and Supplementary Fig. 3B), suggesting that the anti-fibrotic efficacy of both 5′-Azacytidine and Hydralazine is facilitated through de-methylation and subsequent transcriptional re-activated of aberrantly methylated genes, including *Rasal1*.

**Fig. 2:**
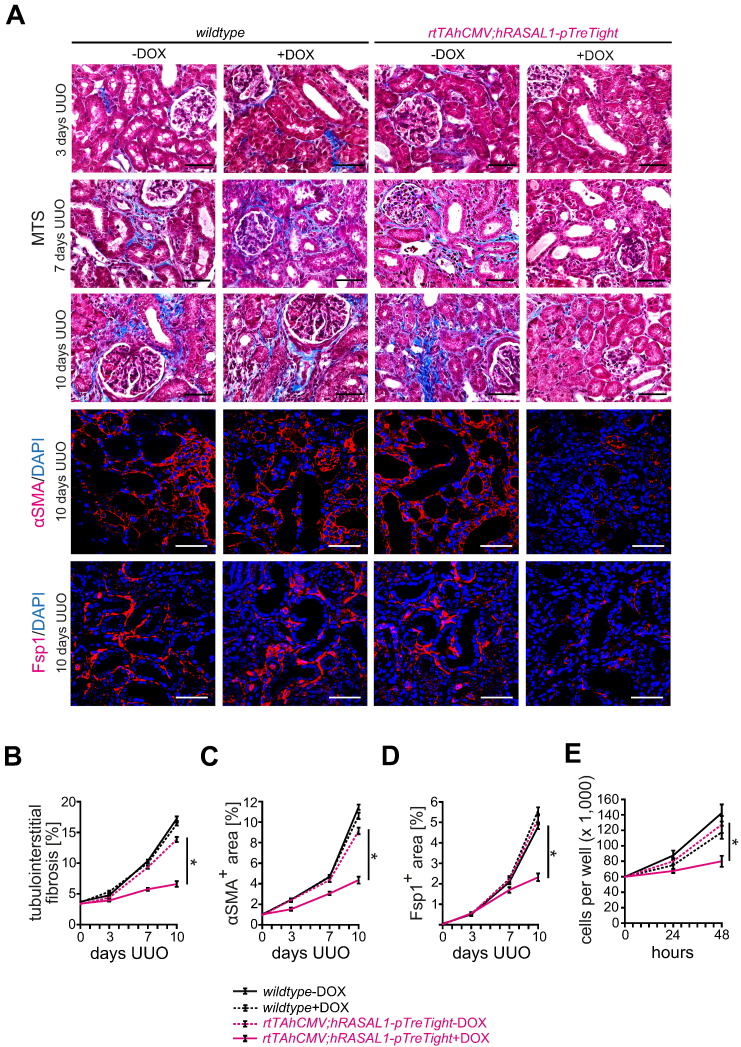
Effect of transgenic RASAL1 over-expression on experimental fibrogenesis and fibroblast accumulation. (A) Representative photomicrographs of Masson's trichrome-stained (MTS), αSMA or Fsp1 immunolabeled kidney sections from *wildtype* and *rtTAhCMV;hRASAL1-pTreTight* mice receiving vehicle buffer PBS (− DOX) or doxycycline (+ DOX). The panels show fibrotic kidney sections from mice challenged with ureteral obstruction (UUO) three, seven and ten days after obstruction. As compared to control mice receiving vehicle buffer PBS (*rtTAhCMV;hRASAL1-pTreTight* − DOX), enhanced RASAL1 expression in induced transgenic mice (*rtTAhCMV;hRASAL1-pTreTight* + DOX) is associated with ameliorated fibrogenesis in the kidney (scale bars: 50 μm). (B–D) The time course summarizes average mean values at the indicated time points of each group (n = 5 in each group, data are presented as means ± s.e.m. *p < 0.05, values of *p* were calculated respective to vehicle-treated *rtTAhCMV;hRASAL1-pTreTight* transgenic mice). (E) Primary fibroblasts were seeded into six-well plates at a density of 60,000 cells per well, trypsinized, and counted after 24 and 48 h. As compared to *wildtype* (*wildtype*-DOX, *wildtype* + DOX) or transgenic fibroblast cultures treated with vehicle buffer PBS (*rtTAhCMV;hRASAL1-pTreTight* − DOX), enhanced RASAL1 expression in induced transgenic fibroblasts (*rtTAhCMV;hRASAL1-pTreTight* + DOX) normalized increased proliferative activity of fibrotic fibroblasts (experiments were replicated four times, data are presented as means ± s.e.m. *p < 0.05, values of *p* were calculated respective to two days vehicle-treated *rtTAhCMV;hRASAL1-pTreTight* fibroblasts).

To further substantiate the link of aberrant *Rasal1* promoter methylation, progressive renal fibrosis and the reno-protective efficacy of de-methylating compounds 5′-Azacytidine and Hydralazine, we next challenged transgenic mice harboring transgenes for doxycycline-inducible RASAL1 over-expression (*rtTAhCMV;hRASAL1-pTreTight*) with UUO (Supplementary Fig. 4A, B). These mice display robust RASAL1 over-expression upon administration of doxycycline and transgene expression is under control of CMV minimal promoter which is devoid of CpG islands and hence resistant to potential transcriptional silencing through promoter methylation when challenged with experimental kidney fibrosis (Supplementary Fig. 4C). When challenged with unilateral ureteral obstruction (UUO), kidneys of *wildtype* and *rtTAhCMV;hRASAL1-pTreTight* mice that did not receive doxycycline (− DOX) developed severe fibrosis within 10 days (Fig. 2A, B). Fibrosis was significantly ameliorated in *rtTAhCMV;hRASAL1-pTreTight* mice in which RASAL1 over-expression was induced in response to doxycycline administration (+ DOX) (Fig. 2A, B). Ameliorated fibrosis in doxycycline-stimulated *rtTAhCMV;hRASAL1-pTreTight* mice (+ DOX) correlated with blunted accumulation of fibroblasts, assessed through immunolabeling using antibodies against αSMA and Fsp1 (Fig. 2A–D), demonstrating that fibroblast activation is attenuated and kidneys are protected when transcriptional suppression of Rasal1 is abolished. In primary fibroblast cultures of *rtTAhCMV;hRASAL1-pTreTight* mice, RASAL1 induction upon doxycycline administration normalized increased intrinsic proliferative activity of fibrotic fibroblasts (Fig. 2E). In summary, we established that Hydralazine is reno-protective in mice when administered at established de-methylating doses, reno-protective efficacy of Hydralazine correlates with amelioration of *Rasal1* promoter methylation, prevention of Rasal1 transcriptional silencing is causally reno-protective and the reno-protective efficacy of Hydralazine is similar to that of the prototypical de-methylating substance 5′-Azacytidine.

### 3.3 Hydralazine De-methylates Rasal1 in Fibrotic Renal Fibroblasts

**Fig. 3:**
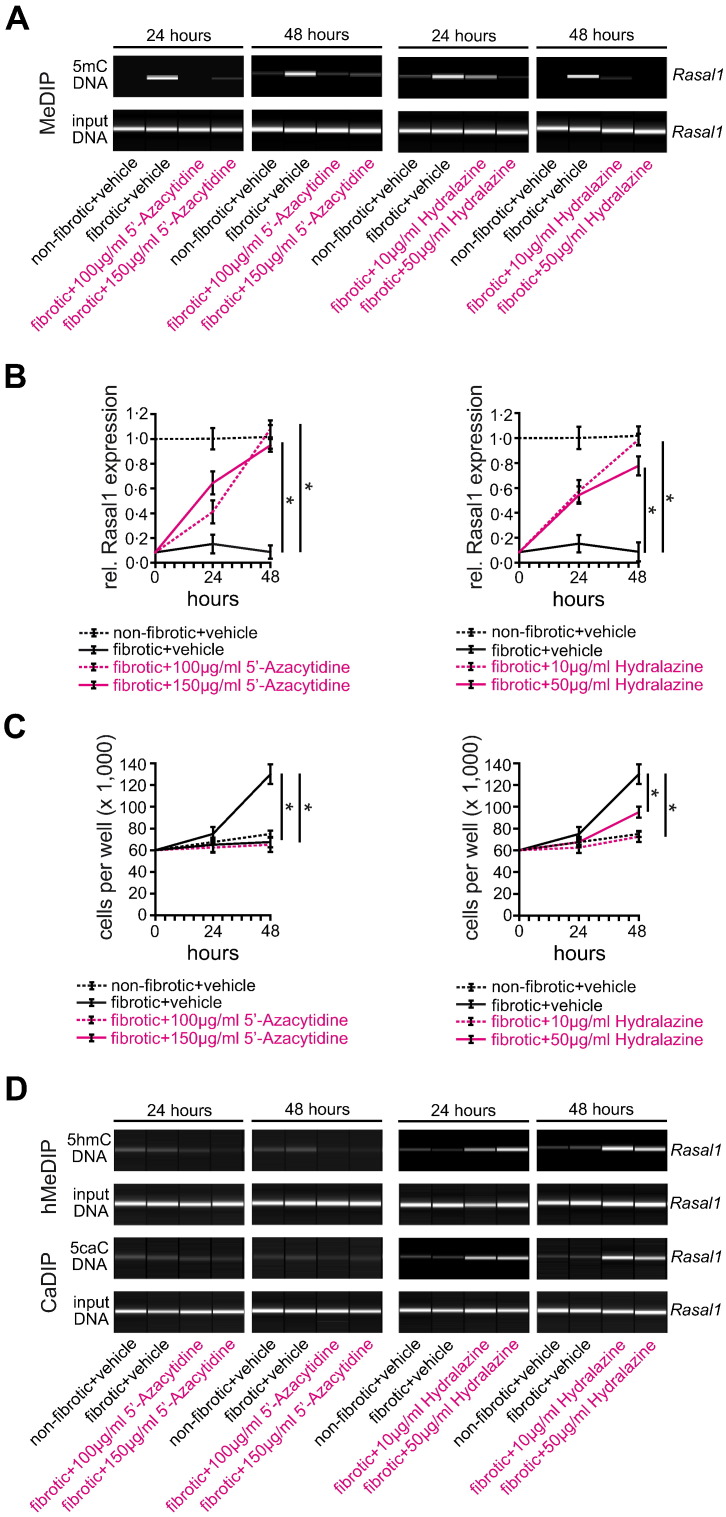
Treatment with de-methylating 5′-Azacytidine or Hydralazine restores Rasal1 mRNA expression due to *Rasal1* promoter de-methylation, involving *Rasal1* promoter hydroxymethylation and carboxylation. (A) Methylation status of the *Rasal1* promoter in primary mouse kidney fibroblasts out of non-fibrotic and fibrotic tissues after treatment with de-methylating 5′-Azacytidine (100 μg/ml or 150 μg/ml) or Hydralazine (10 μg/ml or 50 μg/ml) for 24 and 48 h was analyzed by MeDIP. Treatment normalized aberrant *Rasal1* hypermethylation observed in fibrotic fibroblasts. (B) Rasal1 mRNA expression levels were analyzed by qRT-PCR at time points indicated. Decreased Rasal1 mRNA expression levels observed in fibrotic fibroblast cultures were restored after treatment with de-methylating 5′-Azacytidine or Hydralazine (experiments were done in triplicate, data are presented as means ± s.e.m. *p < 0.05, values of *p* were calculated respective to two days vehicle-treated fibrotic fibroblast cultures). (C) Primary fibroblasts were seeded into six-well plates at a density of 60,000 cells per well, trypsinized and counted after 24 and 48 h. Compared to non-fibrotic fibroblasts, fibrotic fibroblasts are characterized by increased proliferative activity whereas treatment with 5′-Azacytidine or Hydralazine normalized fibrotic fibroblast proliferation (experiments were replicated four times, data are presented as means ± s.e.m. *p < 0.05, values of *p* were calculated respective to two days vehicle-treated fibrotic fibroblast cultures). (D) DNA hydroxymethylation and carboxylation were analyzed by hMeDIP and CaDIP. Whereas none of these modifications were detected in fibrotic fibroblasts upon treatment with 5′-Azacytidine, *Rasal1* promoter de-methylation after Hydralazine treatment involves *Rasal1* hydroxymethylation and *Rasal1* carboxylation indicating active enzymatic DNA de-methylation.

To further substantiate the link between Hydralazine's reno-protective efficacy and its de-methylating activity, we next aimed to explore the mechanisms which underlie its effect on DNA de-methylation. It is well established that the prototypical de-methylating drug 5′-Azacytidine has a bimodal function, as it directly inhibits Dnmt1 (DNA methyltransferase 1, the enzyme which facilitates DNA methylation) and it is incorporated into DNA as cytosine analog prompting DNA repair and replacement of methylated CpGs with naked cytosine (Foulks et al., 2012). In contrast, the mechanisms which underlie de-methylating activity of Hydralazine are still elusive. To gain insights into the de-methylating activity of Hydralazine, we utilized an established cell culture system involving primary renal fibroblasts. We previously described that fibroblasts from fibrotic kidneys (which display an activated phenotype associated with increased autonomous proliferative activity) are characterized by aberrant promoter CpG-island methylation of *Rasal1* (Bechtel et al., 2010, Tampe et al., 2014). We also demonstrated that 5′-Azacytidine effectively de-methylated *Rasal1* and that rescue of endogenous Rasal1 expression normalized proliferative activity of fibrotic renal fibroblasts (to the level of non-fibrotic fibroblasts) (Bechtel et al., 2010, Tampe et al., 2014). Here we demonstrate that the addition of Hydralazine to cell culture media de-methylated *Rasal1* promoter CpG islands (Fig. 3A and Supplementary Fig. 5A, B), associated with re-activation of *Rasal1* expression (Fig. 3B). De-methylating activity was less effective than 5′-Azacytidine, which caused *Rasal1* de-methylation at optimum dosage within 24 h (Fig. 3A), similar to what has been observed in cancer cells (Chuang et al., 2005). In control experiments, neither 5′-Azacytidine nor Hydralazine significantly stimulated Rasal1 expression in non-fibrotic fibroblasts in which *Rasal1* promoter CpG islands are not substantially methylated (Supplementary Fig. 6A, B). Rasal1 re-expression upon 5′-Azacytidine or Hydralazine exposure correlated with normalization of increased proliferative activity of fibrotic renal fibroblasts (Fig. 3C), whereas proliferation of non-fibrotic fibroblasts was not affected by either 5′-Azacytidine or Hydralazine (Supplementary Fig. 6C), establishing that Hydralazine effectively restores Rasal1 expression by inducing de-methylation of its CpG island promoter, resulting in normalization of intrinsic proliferative activity.

We next aimed to gain insights into the mechanisms which underlie Hydralazine-mediated *Rasal1* promoter de-methylation. Because Hydralazine is not incorporated into DNA, we hypothesized that Hydralazine acts by inducing endogenous de-methylating mechanisms. In general, methylated DNA can be de-methylated either through passive or active mechanisms. Passive de-methylation can occur when methylation marks are not copied on the newly synthesized strands meaning that during a first cell division, hemi-methylated DNA is generated and the methylation mark is entirely removed upon the second cell division (Bhutani et al., 2011). For rapid de-methylation to occur, active de-methylation mechanisms involve oxidation of methylated cytosine (5mC) to generate 5-hydroxymethyl-cytosine (5hmC), 5-formyl-cytosine (5fC), and 5-carboxyl-cytosine (5caC) with subsequent base excision and replacement with naked cytosine are required (Bhutani et al., 2011). We observed that *Rasal1* de-methylation upon Hydralazine exposure corresponded with increased *Rasal1* hydroxymethylation and *Rasal1* carboxylation (but not upon exposure to 5′-Azacytidine) (Fig. 3D), suggesting that Hydralazine mediates *Rasal1* de-methylation by active DNA de-methylation involving formation of *Rasal1* hydroxymethylation and *Rasal1* carboxylation. In control experiments, neither 5′-Azacytidine nor Hydralazine significantly stimulated *Rasal1* hydroxymethylation and *Rasal1* carboxylation in non-fibrotic fibroblasts (in which *Rasal1* promoter CpG islands are not substantially methylated) (Supplementary Fig. 6D).

### 3.4 Hydralazine-induced De-methylation Involves Tet3-mediated Hydroxymethylation

**Fig. 4:**
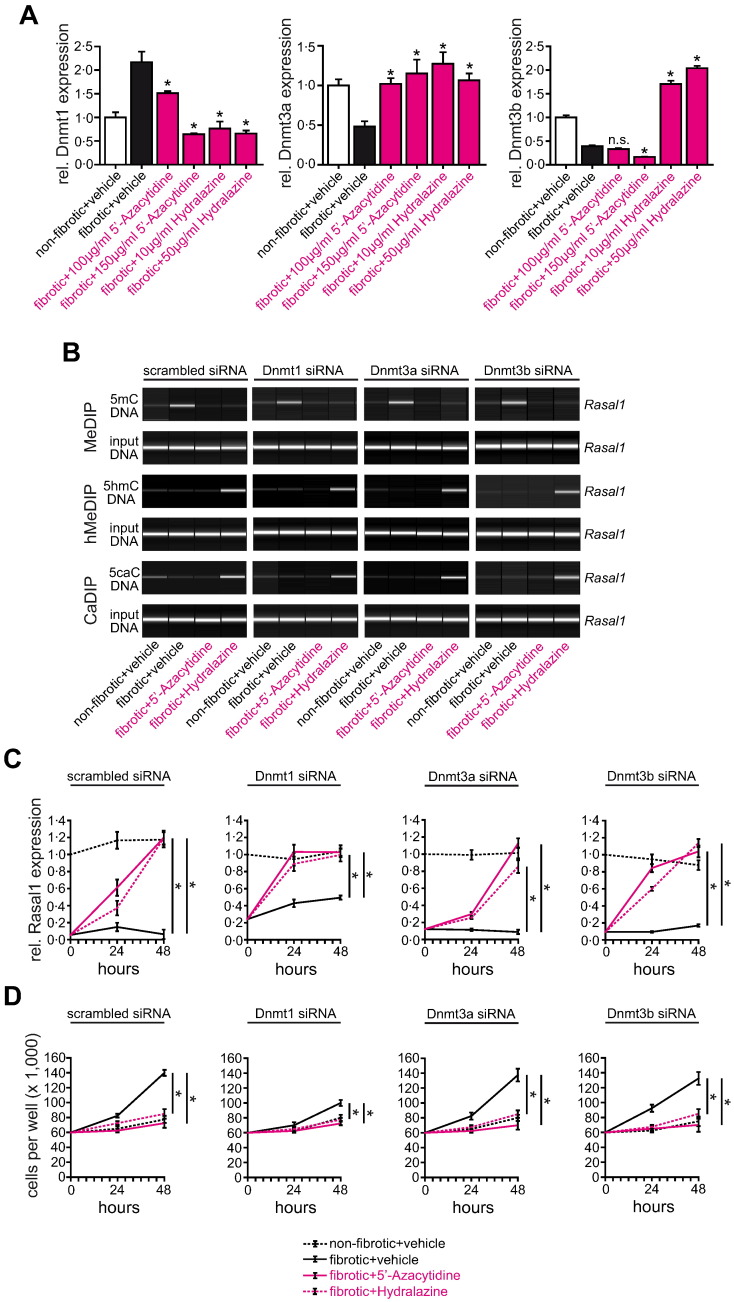
Hydralazine-induced de-methylation of *Rasal1* promoter is mediated through active mechanisms in renal fibroblasts and not exclusively dependent on Dnmt1 inhibition. (A) Analyzed by qRT-PCR, mRNA expression levels of DNA methyltransferases Dnmt1 and Dnmt3a were normalized after treatment with 5′-Azacytidine and Hydralazine (experiments were done in triplicate, data are presented as means ± s.e.m. *p < 0.05, n.s. no significance, values of *p* were calculated respective to vehicle-treated fibrotic cultures). (B) The top pictures show virtual gel images of *Rasal1* PCR products of immunoprecipitated DNA, the bottom pictures show PCR products of input DNA as controls for equal loading in MeDIP, hMeDIP, and CaDIP. Panels display representative analysis of cells which had been transfected with scrambled siRNA (left) or with siRNAs targeting (from left to right) Dnmt1, -3a, or 3b. Knockdown of Dnmts did not mediate rapid *Rasal1* de-methylation as observed upon treatment with Hydralazine suggesting that *Rasal1* de-methylation upon Hydralazine exposure involves mechanisms of active DNA de-methylation. (C) Fibrotic fibroblast cultures were subjected to vehicle buffer, 5′-Azacytidine or Hydralazine, Rasal1 mRNA expression levels were analyzed by qRT-PCR at indicated time points. Decreased Rasal1 mRNA expression levels observed in fibrotic fibroblast cultures were restored after treatment with de-methylating 5′-Azacytidine or Hydralazine. Knockdown of Dnmts did not affect Rasal1 mRNA expression levels (experiments were done in triplicate, data are presented as means ± s.e.m. *p < 0.05, values of *p* were calculated respective to two days vehicle-treated fibrotic fibroblast cultures). (D) Depletion of Dnmt1, -3a, or -3b did not affect proliferative activity of fibrotic fibroblasts, 5′-Azacytidine and Hydralazine were still capable to normalize intrinsic proliferative activity (experiments were replicated four times, data are presented as means ± s.e.m. *p < 0.05, values of *p* were calculated respective to two days vehicle-treated cultures).

To gain insights into the de-methylating activity of Hydralazine, we first analyzed expression levels of DNA methyltransferases (Dnmts) which facilitate CpG island methylation. Hydralazine significantly reduced Dnmt1 mRNA and protein expression levels (Fig. 4A and Supplementary Fig. 7A), whereas Dnmt3a and Dnmt3b were increased upon Hydralazine exposure (Fig. 4A). However, knockdown of Dnmts did not mediate *Rasal1* de-methylation as observed upon treatment with Hydralazine (Fig. 4B and Supplementary Fig. 8A). When Dnmts were depleted, Hydralazine was still capable of inducing *Rasal1* hydroxymethylation and carboxylation (Fig. 4B), which was associated with restoration of Rasal1 expression levels (Fig. 4C), and normalization of increased proliferative activity of fibrotic fibroblasts (Fig. 4D), suggesting that Hydralazine-induced de-methylation is mediated through active mechanisms in renal fibroblasts and is not exclusively dependent on Dnmt1 inhibition.

**Fig. 5:**
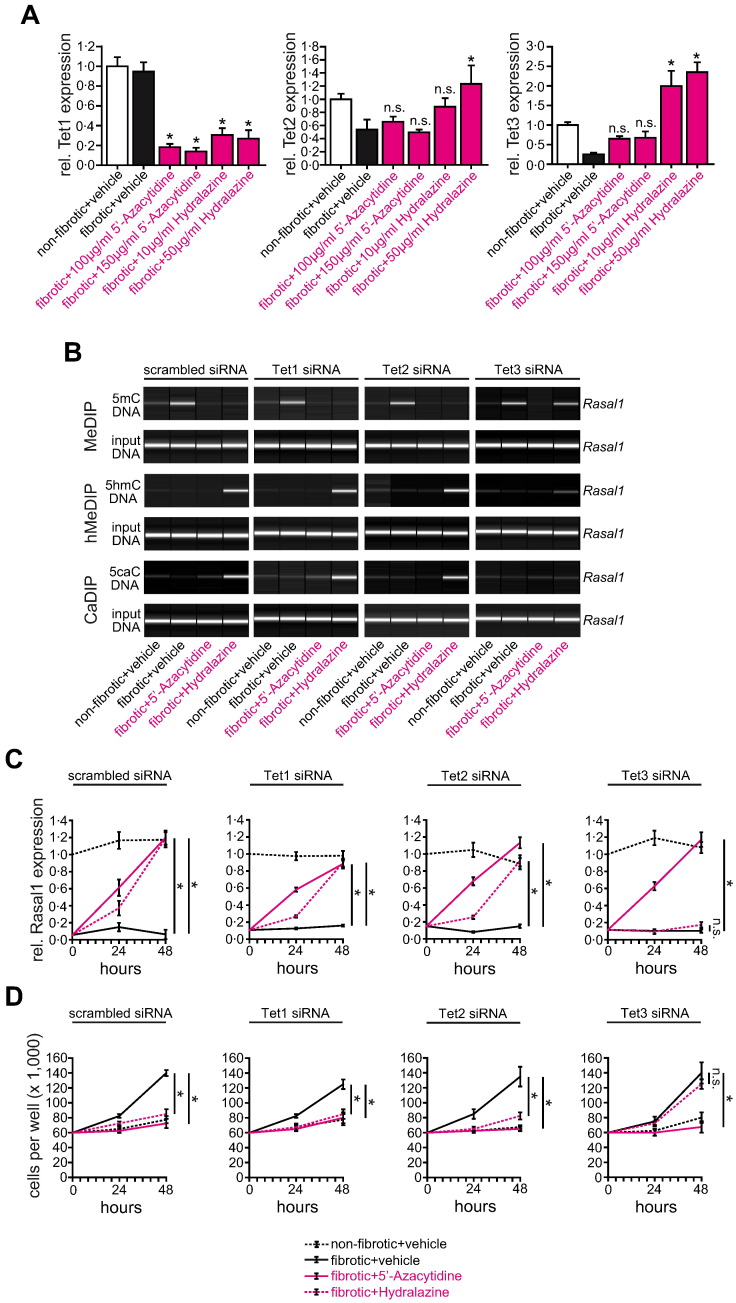
Normalization of *Rasal1* promoter methylation upon de-methylating Hydralazine involves DNA hydroxylase Tet3. (A) As analyzed by qRT-PCR, Hydralazine treatment is associated with induction of DNA hydroxylase Tet3 in primary fibrotic fibroblast cultures (experiments were done in triplicate, data are presented as means ± s.e.m. *p < 0.05, n.s. no significance, values of *p* were calculated respective to vehicle-treated fibrotic cultures). (B) Panels display representative analysis of cells which had been transfected with scrambled siRNA (left) or with siRNAs targeting (from left to right) Tet1, -2, or -3. Whereas neither knockdown of Tet1 nor Tet2 affected the efficacy of Hydralazine to induce *Rasal1* promoter de-methylation, *Rasal1* hydroxymethylation, or *Rasal1* carboxylation, depletion of Tet3 resulted in failure of Hydralazine to normalize *Rasal1* methylation due to inefficacy of *Rasal1* hydroxymethylation and *Rasal1* carboxylation formation. (C) Fibrotic fibroblast cultures were subjected to vehicle buffer PBS, 5′-Azacytidine, or Hydralazine, Rasal1 mRNA expression levels were analyzed by qRT-PCR at indicated time points. Depletion of Tet1 and Tet2 did not affect the capacity of Hydralazine to restore Rasal1 mRNA expression. In contrast, Hydralazine no longer normalized decreased Rasal1 expression levels observed in fibrotic fibroblasts when Tet3 was depleted (experiments were done in triplicate, data are presented as means ± s.e.m. *p < 0.05, n.s. no significance, values of *p* were calculated respective to vehicle-treated fibrotic cultures). (D) Consistent with failure of Hydralazine to normalize *Rasal1* promoter methylation and mRNA expression after Tet3 depletion, Hydralazine no longer decreased the proliferative activity of fibrotic fibroblast cultures (experiments were replicated four times, data are presented as means ± s.e.m. *p < 0.05, n.s. no significance, values of *p* were calculated respective to two days vehicle-treated cultures).

Because *Rasal1* de-methylation upon Hydralazine exposure corresponded with increased *Rasal1* hydroxymethylation (Fig. 3D), we hypothesized that Hydralazine involved oxidation of methylated cytosine through Tet proteins (Ten-Eleven Translocation enzymes) to generate hydroxymethylated cytosine, we next analyzed the effect of Hydralazine on expression levels of Tet1, Tet2, and Tet3 (Tahiliani et al., 2009, Ito et al., 2010). Of the three known Tets, we observed that only Tet3 expression was induced upon Hydralazine exposure (Fig. 5A and Supplementary Fig. 7B), siRNA-mediated depletion of Tet3 prevented Hydralazine-induced *Rasal1* de-methylation (Fig. 5B and Supplementary Fig. 8B), restoration of mRNA expression levels (Fig. 5C), and normalization of fibrotic fibroblast proliferation (Fig. 5D) in response to Hydralazine, while Tet1 or Tet2 knockdown had no effects (Fig. 5B–D and Supplementary Fig. 8B). Neither Dnmt nor Tet depletion affected *Rasal1* de-methylation or transcriptional restoration upon exposure to 5′-Azacytidine (Figs. 4B, C and 5B, C).

**Fig. 6:**
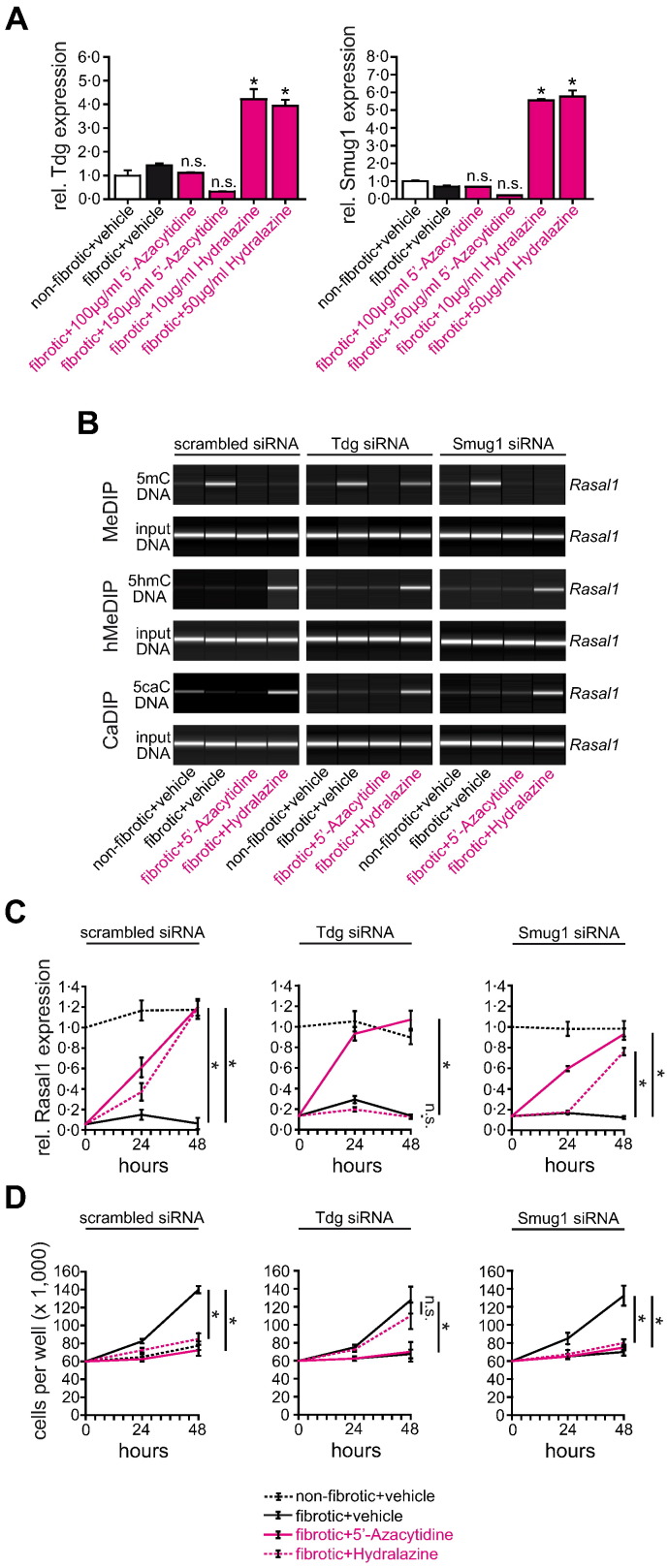
Normalization of *Rasal1* methylation by Hydralazine involves DNA glycosylase Tdg. (A) As analyzed by qRT-PCR, Hydralazine administration is associated with induction of DNA glycosylases Tdg and Smug 1 (experiments were done in triplicate, data are presented as means ± s.e.m. *p < 0.05, n.s. no significance, values of *p* were calculated respective to vehicle-treated fibrotic cultures). (B) Cells had been transfected with scrambled siRNA (left) or with siRNAs targeting Tdg (middle) or Smug1 (right). Knockdown of Tdg resulted in impaired *Rasal1* promoter de-methylation upon Hydralazine treatment whereas depletion of Smug1 did not affect normalization of *Rasal1* promoter methylation. (C) Whereas knockdown of Smug1 did not affect normalization of Rasal1 mRNA expression upon Hydralazine treatment, Hydralazine failed to restore Rasal1 expression levels when Tdg was depleted (experiments were done in triplicate, data are presented as means ± s.e.m. *p < 0.05, n.s. no significance, values of *p* were calculated respective to vehicle-treated fibrotic cultures). (D) Consistent with insufficient normalization of *Rasal1* promoter methylation and mRNA expression after Tdg depletion, Hydralazine no longer decreased the proliferative activity of fibrotic fibroblast cultures (experiments were replicated four times, data are presented as means ± s.e.m. *p < 0.05, n.s. no significance, values of *p* were calculated respective to two days vehicle-treated cultures).

Because our data suggested that Hydralazine causes *Rasal1* de-methylation through a mechanism involving Tet3-mediated hydroxymethylation, we next elucidated the involvement of additional factors which are involved in replacement of hydroxymethylated cytosines with naked cytosine bases. In germline cells, hydroxymethylated cytosine is further oxidized to 5-formyl-cytosine (5fC) and 5-carboxyl-cytosine (5caC) before excision of 5caC by DNA glycosylase Tdg or Smug1, and restoration of cytosine via follow-on base excision repair (Bhutani et al., 2011). Alternatively, 5hmC is deaminated by the Aid/Apobec family of cytidine deaminases, before Tdg-mediated base excision repair (Bhutani et al., 2011). While upon exposure to Hydralazine, neither expression of deaminases Aicda, Apobec1, or Apobec3 was significantly regulated (Supplementary Fig. 7C), Tdg and Smug1 expression (which facilitate base excision repair) were significantly increased upon Hydralazine treatment (Fig. 6A and Supplementary Fig. 7D). Only siRNA-mediated depletion of Tdg effectively prevented *Rasal1* de-methylation (Fig. 6B and Supplementary Fig. 8C), mRNA restoration (Fig. 6C), and normalization of fibrotic fibroblast proliferation (Fig. 6D), suggesting that Hydralazine involves Tdg-mediated base excision repair in facilitating *Rasal1* de-methylation. In summary, Hydralazine re-activated expression of Rasal1 in fibrotic renal fibroblasts through a mechanism involving Tet3-mediated *Rasal1* hydroxymethylation, Tdg-mediated excision, and subsequent replacement with naked cytosine. Albeit the de-methylating activity of Hydralazine was inferior to that of 5′-Azacytidine, it was sufficient enough to re-activate Rasal1 expression and normalize intrinsic proliferative activity of fibrotic renal fibroblasts.

### 3.5 Evidence for Tet3-mediated Rasal1 De-methylation in Hydralazine-treated Mice

**Fig. 7:**
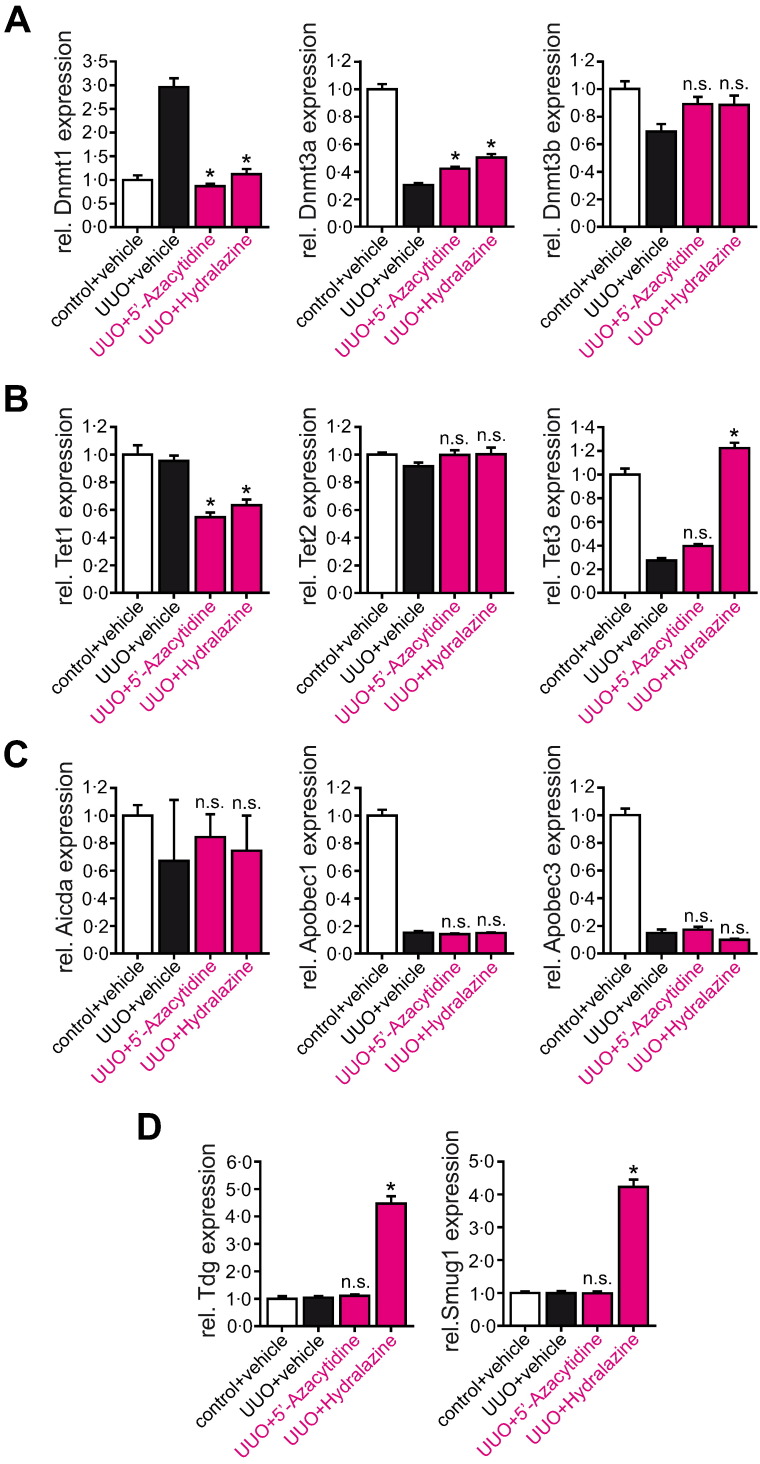
Impact of 5′-Azacytidine and Hydralazine on mRNA expression levels of genes involved in CpG methylation and de-methylation in vivo. (A–D) Mice were challenged with unilateral ureteral obstruction (UUO) and treated with either vehicle buffer PBS, 5′-Azacytidine, or Hydralazine (10 mg/kg/day or 5 mg/kg/day, respectively) and sacrificed ten days after ureteral obstruction when kidneys had developed severe tubulointerstitial fibrosis. mRNA expression levels of Dnmt1 and Dnmt3a were normalized after treatment with 5′-Azacytidine and Hydralazine. Among Tet hydroxylases, Hydralazine induced expression of Tet3, but not of Tet1 or Tet2, whereas 5′-Azacytidine did not increase expression of either Tet gene. Hydralazine treatment had no effect on expression levels of cytosine deaminases Aicda, Apobec1, or Apobec3, whereas DNA glycosylases Tdg and Smug 1 were induced after Hydralazine administration in UUO mice, 5′-Azacytidine did not impact mRNA expression levels (experiments were done in triplicate, data are presented as means ± s.e.m. *p < 0.05, n.s. no significance, values of *p* were calculated respective to vehicle-treated UUO mice).

**Fig. 8:**
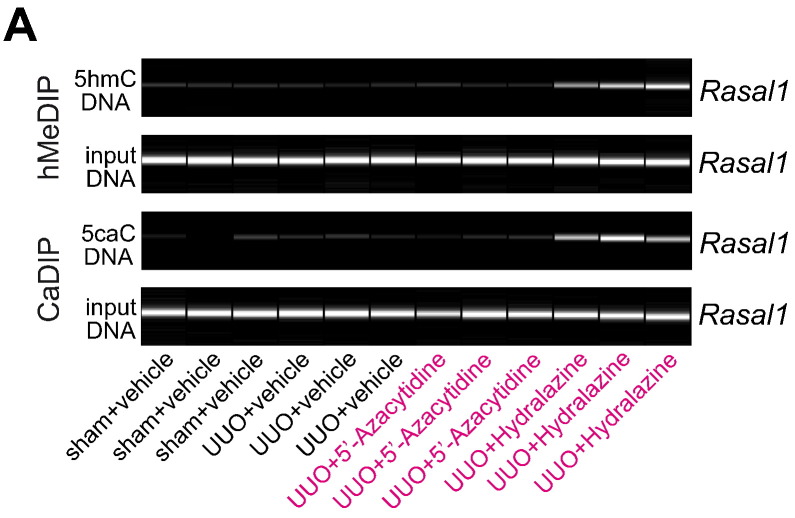
Normalization of *Rasal1* promoter methylation upon Hydralazine involves enzymatic de-methylation dependent on cytosine hydroxymethylation and carboxylation. (A) We performed DNA immunoprecipitations on hydroxymethylated (hMeDIP) and carboxylated cytosines (CaDIP) to assess the effect of 5′-Azacytidine and Hydralazine on enzymatic cytosine modifications involved in active DNA de-methylation in mice challenged with unilateral ureteral obstruction (UUO). Whereas none of these modifications were observed in fibrotic mice treated with either vehicle buffer PBS (UUO + vehicle) or 5′-Azacytidine (UUO + 5′-Azacytidine), treatment with Hydralazine (UUO + Hydralazine) was associated with *Rasal1* hydroxymethylation and *Rasal1* carboxylation, suggesting that de-methylating Hydralazine involves enzymatic modifications in normalization of *Rasal1* promoter methylation.

We next explored if the identified pathway was similarly involved when kidneys were protected from experimental fibrogenesis upon Hydralazine administration. We analyzed expression of Dnmts, Tets, Aicda, Apobecs, Tdg, and Smug1 by qRT-PCR in kidney lysates of mice which had been challenged with ureteral obstruction (Fig. 7A–D, compare to Fig. 1) or folic acid (Supplementary Fig. 9A–D, compare to Supplementary Fig. 1). In kidneys of mice that were challenged with UUO, blunted fibrosis upon either 5′-Azacytidine or Hydralazine administration correlated with suppressed Dnmt1 expression (Fig. 7A). Among Tet hydroxylases, Hydralazine induced expression of Tet3, but not of Tet1 or Tet2, whereas 5′-Azacytidine did not increase expression of either Tet gene (Fig. 7B). Similar to results observed in cell culture, administration of Hydralazine had no effect on expression levels of cytosine deaminases Aicda, Apobec1, or Apobec3 (Fig. 7C), whereas DNA glycosylases Tdg and Smug1 were induced after Hydralazine administration in UUO mice (Fig. 7D). Induction of the Tet3/Tdg cascade correlated with increased *Rasal1* hydroxymethylation and *Rasal1* carboxylation (Fig. 8A), suggesting that mechanisms which mediate *Rasal1* de-methylation in Hydralazine-treated mice correspond to Hydralazine-induced de-methylation in cultured renal fibroblasts. Evidence for Tet3/Tdg-mediated de-methylation of *Rasal1* was similarly observed in folic acid (FA)-challenged *CD1* mice (Supplementary Figs. 9A–D and 10A). In summary, our studies suggest that Hydralazine ameliorates experimental renal fibrosis through inducing de-methylation of aberrantly methylated *Rasal1* (and additional genes as well) by Dnmt1 inhibition and by inducing the endogenous Tet3/Tdg de-methylation pathway.

### 3.6 Circulating Methylated RASAL1 CpG Island Promoter Fragments Are Reduced in Patients on Dihydralazine Medication

**Fig. 9:**
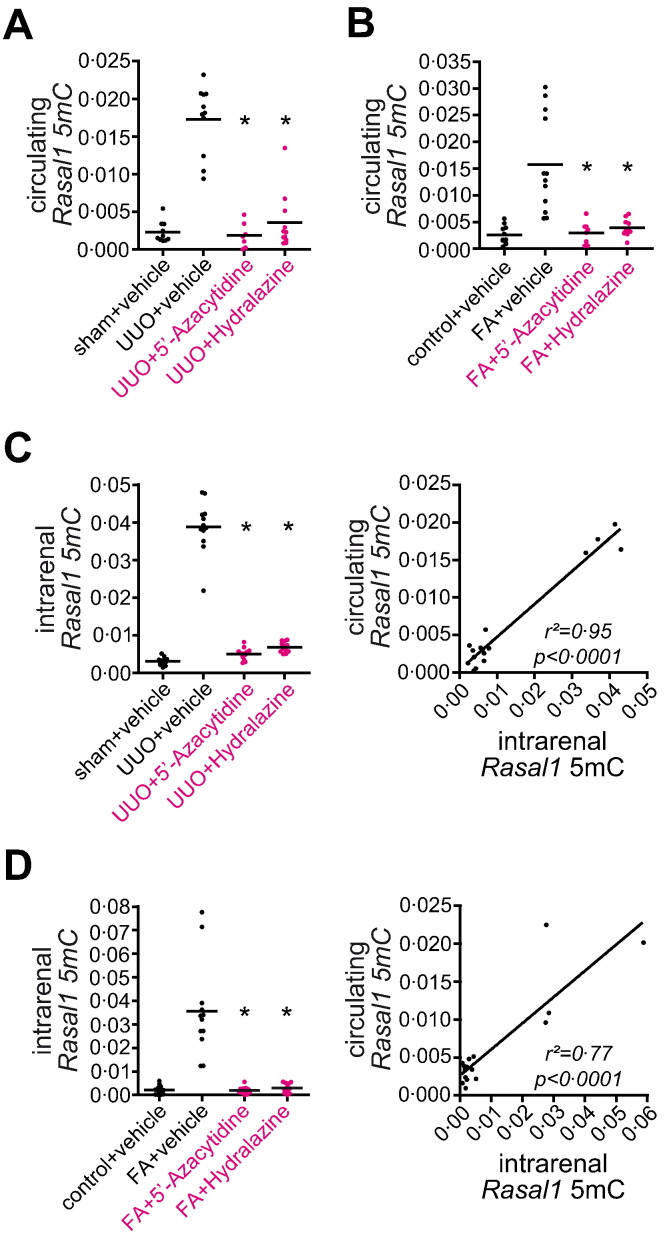
Circulating *Rasal1* promoter DNA fragments correlate with intrarenal *Rasal1* promoter methylation and kidney fibrosis in mouse models of experimental fibrogenesis. (A–D) Experimental renal fibrogenesis is associated with increased intrarenal *Rasal1* methylation and circulating *Rasal1* promoter DNA fragments. Mice challenged with unilateral ureteral obstruction (UUO) or folic acid-induced nephropathy (FA) were treated with either vehicle buffer PBS, 5′-Azacytidine, or Hydralazine (10 mg/kg/day or 5 mg/kg/day, respectively) and methylated DNA was isolated from kidney and correlated blood samples using methylated DNA immunoprecipitation (MeDIP). Compared to healthy control mice, kidney fibrosis in both mouse models used was associated with increased intrarenal *Rasal1* methylation and circulating methylated *Rasal1* promoter DNA fragments, whereas treatment with anti-fibrotic and de-methylating 5′-Azacytidine or Hydralazine normalized intrarenal *Rasal1* methylation associated with decreased circulating methylated *Rasal1* DNA fragments. Circulating methylated *Rasal1* promoter DNA fragments reflected intrarenal *Rasal1* methylation in mice challenged with experimental kidney fibrosis (experiments were done in triplicate, data are presented as scatter dot blots with lines at means. *p < 0.05, values of *p* were calculated respective to vehicle-treated fibrotic mice, *r*^2^ and *p* value are presented in the correlation analysis).

Our findings that *Rasal1* promoter CpG island methylation contributed to progression of renal fibrosis, Hydralazine ameliorated aberrant promoter methylation, and blunted renal fibrosis and intrarenal *Rasal1* promoter methylation levels correlate with Hydralazine therapy in mice offered the attractive combination of a clinically approved drug (Hydralazine and its derivate Dihydralazine) and a potential biomarker in human patients (*RASAL1* promoter methylation). However, the need for a renal biopsy to assess intrarenal degree of *RASAL1* methylation appeared prohibitive in this regard. Based on several studies which demonstrated that aberrant CpG island promoter methylation within tumors is reflected by circulating methylated promoter fragments, we hypothesized that aberrant CpG island promoter methylation associated with renal fibrosis could be similarly correlated with increased levels of circulating DNA fragments (Schwarzenbach et al., 2011). Analysis of circulating *Rasal1* promoter DNA fragments in peripheral blood from mice which had been challenged with either ureteral obstruction (UUO) or folic acid (FA) and had received vehicle buffer PBS, 5′-Azacytidine, or Hydralazine demonstrated that levels of circulating methylated *Rasal1* promoter DNA fragments were significantly increased in mice with kidney fibrosis due to challenge with either UUO or FA as compared to control mice receiving vehicle-buffer PBS (Fig. 9A, B, compare to Fig. 1 and Supplementary Fig. 1). In mice with ameliorated fibrosis due to treatment with de-methylating agent 5′-Azacytidine or Hydralazine, levels of circulating *Rasal1* promoter DNA fragments were significantly reduced when compared to fibrotic untreated mice (Fig. 9A, B), correlating with reduced fibrosis (compare to Fig. 1 and Supplementary Fig. 1). Circulating levels of methylated *Rasal1* promoter DNA fragments correlated with degree of intrarenal *Rasal1* promoter CpG island methylation (Fig. 9C, D). From this data we concluded that circulating *Rasal1* promoter DNA fragments correlate with intrarenal *Rasal1* promoter CpG island methylation, and possibly with extent of fibrosis in experimental renal fibrosis as it does in cancer patients. We also concluded that longitudinal analysis of circulating methylated *Rasal1* promoter DNA fragments reflected therapeutic efficacy of de-methylating agents 5′-Azacytidine and Hydralazine.

**Fig. 10:**
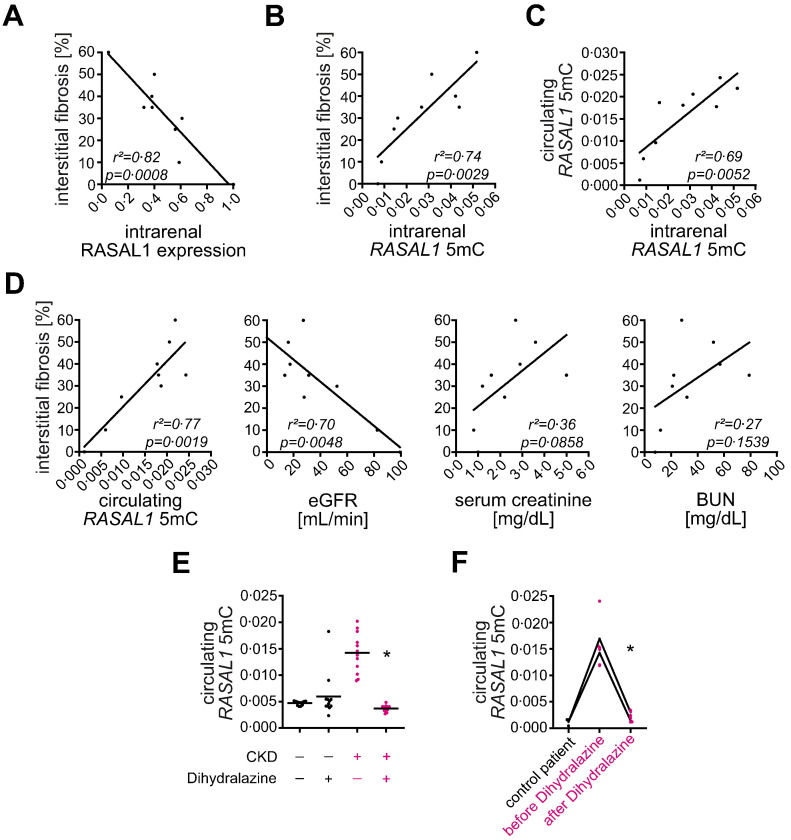
Circulating *RASAL1* promoter DNA fragments correlate with intrarenal *RASAL1* promoter methylation and degree of fibrosis in CKD patients irrespective of the underlying disease. (A) Kidney fibrosis negatively correlated with endogenous RASAL1 mRNA expression levels (experiments were done in triplicate, *r^2^* and *p* value are presented in the correlation analysis). (B, C) Extent of interstitial fibrosis is positively correlated with intrarenal *RASAL1* promoter methylation associated with increased levels of circulating *RASAL1* promoter DNA fragments (experiments were done in triplicate, *r^2^* and *p* value are presented in the correlation analysis). (D) Compared to conventional parameters of renal function estimated glomerular filtration rate (eGFR), serum creatinine, or blood urea nitrogen (BUN), circulating methylated *RASAL1* promoter DNA fragments correlated best with extent of tubulointerstitial fibrosis of corresponding patients (*r^2^* and *p* values are presented in the corresponding correlation analysis). (E) In hypertensive patients with CKD (right panels in pink), Dihydralazine medication was associated with normalization of circulating methylated *RASAL1* promoter DNA fragments (n = 4 in each group, experiments were done in triplicate, data are presented as scatter dot blots with lines at means. *p < 0.05, value of *p* was calculated respective to patients with HTN and CKD without Dihydralazine medication). (F) Increased circulating methylated *RASAL1* promoter DNA fragment levels in two CKD patients were normalized one week after initiation of Dihydralazine treatment (experiments were done in triplicate, data are presented as scatter dot blots with connection lines of means. *p < 0.05, value of *p* was calculated respective to analysis before initiation of Dihydralazine medication).

We next explored the possibility that circulating methylated *RASAL1* promoter fragments could similarly reflect intrarenal *RASAL1* methylation (and possibly fibrosis) in patients with CKD as they do in mice. We analyzed kidney biopsy specimens and matching peripheral blood samples from a small cohort of patients with varying degree of renal fibrosis at time of biopsy (Supplementary Table D). Renal fibrosis was inversely correlated with intrarenal RASAL1 mRNA expression levels (Fig. 10A) and the degree of intrarenal *RASAL1* promoter CpG island methylation correlated with the degree of renal fibrosis, irrespective of the underlying disease (Fig. 10B), validating previous studies (Tampe et al., 2014). Analysis of circulating methylated *RASAL1* promoter DNA fragments demonstrated that levels of circulating methylated *RASAL1* promoter fragments reflect intrarenal *RASAL1* promoter CpG island methylation (Fig. 10C). In this cohort, positive correlation between circulating *RASAL1* promoter fragment levels and degree of interstitial fibrosis at the time of biopsy was superior to that of estimated glomerular filtration rate (eGFR), serum creatinine, or blood urea nitrogen (BUN) levels (Fig. 10D).

We next aimed to gain insights into the de-methylating activity of Dihydralazine in patients with CKD. For this purpose we compared levels of circulating methylated *RASAL1* promoter DNA fragments in patients with comparable stage of chronic kidney disease due to malignant arterial hypertension (HTN) with or without Dihydralazine treatment (Supplementary Table E). Levels of circulating *RASAL1* promoter DNA fragments were significantly decreased in patients on Dihydralazine medication as compared to patients who did not receive Dihydralazine (Fig. 10E). In two CKD patients who were newly put on Dihydralazine medication due to therapy-resistant arterial HTN (Supplementary Table F), we observed decreased levels of circulating *RASAL1* promoter DNA fragments within one week of Dihydralazine treatment (Fig. 10F), providing evidence that lower levels of circulating *RASAL1* promoter DNA fragments in the Dihydralazine group were a direct reflection of Dihydralazine's de-methylating activity.

### 3.7 Evidence for Dose-dependency of Reno-protective Effect of Hydralazine

**Fig. 11:**
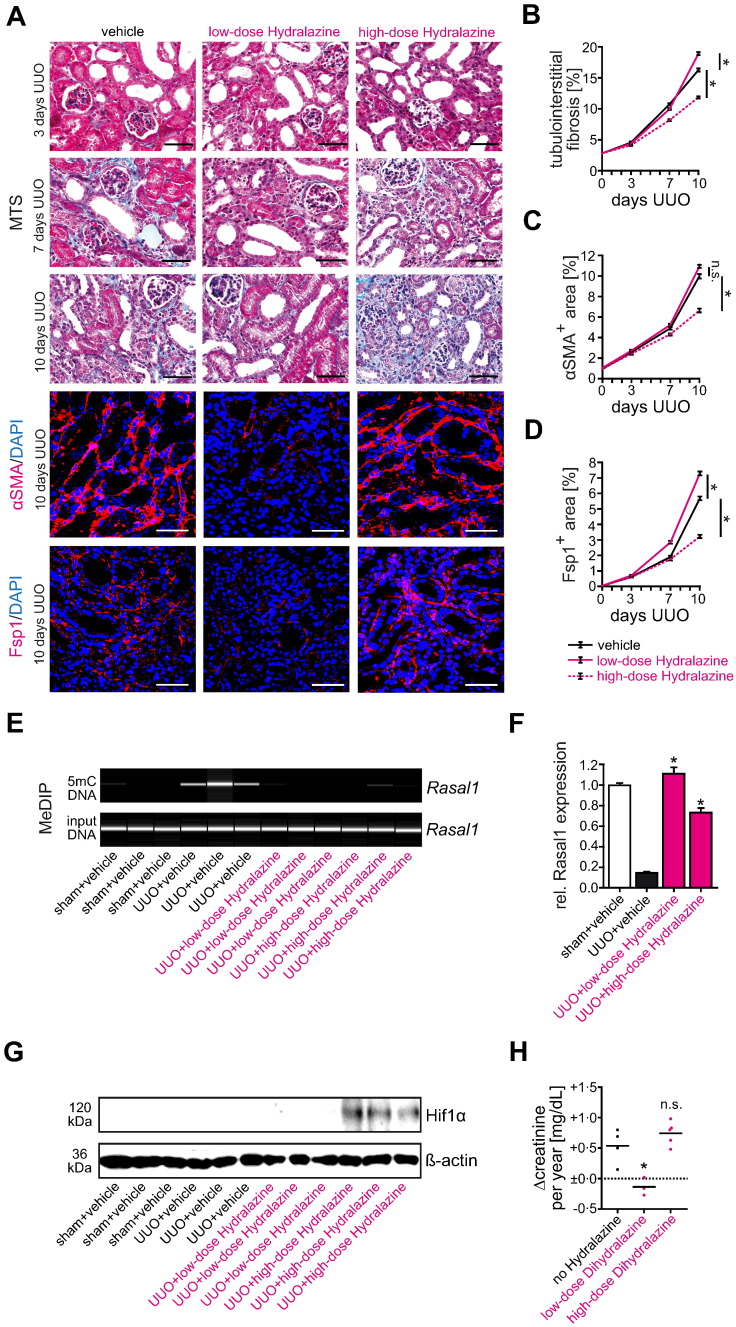
Comparison of low-dose and high-dose Hydralazine administration in experimental kidney fibrosis and CKD patients. (A) Representative photomicrographs of Masson's trichrome-stained (MTS), αSMA or Fsp1 immunolabeled kidney sections from mice receiving either vehicle buffer PBS, low-dose (5 mg/kg/day) or high-dose Hydralazine (50 mg/kg/day). The panel shows fibrotic kidney sections from mice challenged with UUO at indicated time points after ureteral obstruction. As compared to control mice receiving vehicle buffer PBS, only treatment with low-dose Hydralazine attenuated renal fibrogenesis and fibroblast accumulation. In contrast, high-dose Hydralazine failed to attenuate kidney fibrosis in this mouse model (scale bars: 50 μm). (B–D) The time course summarizes average values at the indicated time points of each group (n = 6 in each group, data are presented as means ± s.e.m. *p < 0.05, values of *p* were calculated respective to vehicle-treated UUO mice). (E, F) *Rasal1* promoter methylation was analyzed by MeDIP in mice challenged with unilateral ureteral obstruction (UUO) and treated with either vehicle buffer PBS, 5 mg/kg/day (low-dose), or 50 mg/kg/day Hydralazine (high-dose), Rasal1 mRNA expression levels were analyzed by qRT-PCR. As compared to vehicle-treated UUO mice, both concentrations of Hydralazine were capable to normalize *Rasal1* promoter methylation and mRNA expression (n = 6 in each group, experiments were done in triplicate, data are presented as means ± s.e.m. *p < 0.05, values of *p* were calculated respective to vehicle-treated UUO mice). (G) As determined by SDS-Page of total kidney protein lysates and subsequent immunoblotting, we found levels of Hif1α markedly increased in kidneys of mice that received high-dose Hydralazine at 50 mg/kg/day, but not in kidneys of mice which had received Hydralazine at 5 mg/kg/day (n = 3 in each group). (H) Compared to hypertensive patients without Dihydralazine medication (n = 4), low-dose Dihydralazine medication was associated with attenuation of CKD progression indicated by blunted rise of serum creatinine from baseline over time (n = 3). In contrast, high doses of Dihydralazine failed to attenuate CKD progression (n = 5, data are presented as scatter dot blots with lines at means. *p < 0.05, n.s. no significance, values of *p* were calculated respective to patients without Dihydralazine medication).

Comparison of our promising findings in mice with existing literature revealed that the Hydralazine dose which we used in our animal studies, and which had been established as an optimum de-methylating dose in previous cancer studies (5 mg/kg/day), does not lower blood pressure and was below the anti-hypertensive dose commonly used in mice (50 mg/kg applied up to four times daily). To enable better comparison of our studies with the bulk of existing literature, we performed an additional set of experiments in which we challenged mice with UUO (causing fibrosis in the obstructed kidney without hypertension), administered Hydralazine at doses of either 5 mg/kg/day or 50 mg/kg/day and collected kidneys for analyses after three, seven, and ten days of ureteral obstruction for tissue analysis. While degree of fibrosis and fibroblast accumulation was significantly improved in mice that received low-dose Hydralazine at 5 mg/kg/day as compared to mice that received vehicle buffer PBS, we did not observe significant protection from fibrosis in mice that received high-dose Hydralazine at 50 mg/kg/day (Fig. 11A–D). Because *Rasal1* methylation and transcriptional silencing were equally blunted in kidneys of mice which had received either 5 mg/kg/day or 50 mg/kg/day Hydralazine (Fig. 11E, F), we hypothesized that additional adverse effects, independent of CpG island promoter de-methylation, might be induced in a dose-dependent manner. In this regard, previous studies suggested that Hydralazine could directly enhance intracellular levels of Hypoxia-inducible factor 1-α (Hif1α), a well-established enhancer of chronic progressive kidney disease (Knowles et al., 2004). Therefore we analyzed Hif1α in our cohorts of Hydralazine-treated UUO mice by SDS-Page of total kidney protein lysates and subsequent immunoblotting and found levels of Hif1α markedly increased in kidneys of mice that received high-dose Hydralazine at 50 mg/kg/day, but not in kidneys of mice which had received Hydralazine at 5 mg/kg/day (Fig. 11G).

We next aimed to gain possible insights into the reno-protective efficacy of low-dose Hydralazine in patients. For this purpose, we performed retrospective analysis on patients compared to patients with impaired kidney function (CKD3) due to malignant hypertension and we compared patients who were maintained on low-dose Dihydralazine (12.5 to 25 mg/day) over more than 12 months to patients on standard anti-hypertensive Dihydralazine regimen (100 to 200 mg/day) and with a cohort of patients without anti-hypertensive Dihydralazine treatment (Supplementary Table G). While serum creatinine levels increased over 12 months at typical rates, Dihydralazine given at standard regimen had no effect, whereas increase of serum creatinine levels was significantly blunted in those patients in which Dihydralazine had been maintained at low dosages independent of impact on blood pressure mirroring our observations in mice (Fig. 11H and Supplementary Fig. 11A, B).

## 4 Discussion

**Fig. 12:**
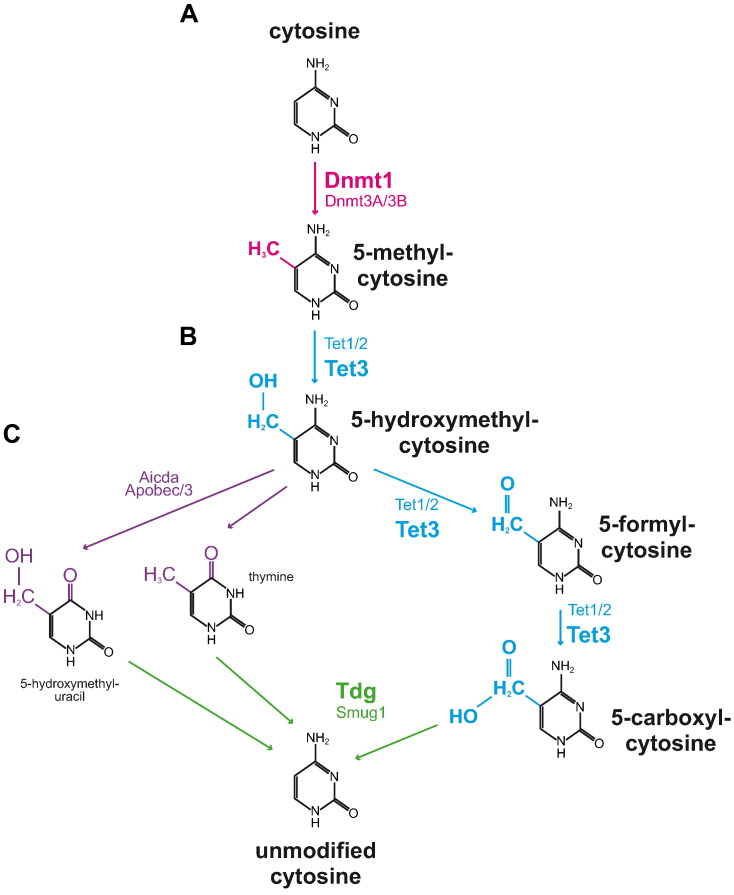
Schematic illustration of enzymes involved in cytosine methylation/de-methylation. (A) Methylation of cytosine bases to 5-methyl-cytosine is mediated by DNA methyltransferases Dnmt1, -3a, and 3b. Hydralazine treatment is associated with Dnmt1 inhibition. (B) 5-Methyl-cytosine bases are converted to 5-hydroxymethyl-cytosines by DNA hydroxylase members Tet1, -2 and, -3. Formation of 5-hydroxymethyl-cytosine upon Hydralazine treatment is Tet3-dependent. (C) The cytidine deaminases Aicda, Apobec1, and -3 convert 5-hydroxymethyl-cytosine to 5-hydroxymethyl-uracil or thymine which is replaced by naked cytosine bases mediated by DNA glycosylases Tdg and Smug1. Alternatively, 5-hydroxymethyl-cytosine bases can be converted to 5-formyl-cytosine and 5-carboxyl-cytosine by Tet1, -2, and -3 and naked cytosine is generated by DNA glycosylases Tdg and Smug1. Treatment with de-methylating Hydralazine is associated with Tet3-induced formation of 5-formyl-cytosine and 5-carboxyl-cytosine, subsequent excision by Tdg, and replacement with unmodified cytosine.

This study adds further evidence for a contribution of aberrant promoter CpG island methylation to the progression of renal fibrogenesis and its potential as a biomarker and therapeutic target. While there are likely additional genes affected by CpG island promoter methylation, this study provides mechanistic evidence for the first time that aberrant transcriptional silencing of *Rasal1* causally contributes to progression of renal fibrogenesis and that *Rasal1* methylation is not just part of an unspecific methylation signature in renal fibrosis. In this regard, normalization of aberrant promoter methylation through administration of 5′-Azacytidine or Hydralazine was associated with attenuated fibroblast activation and fibrogenesis in experimental fibrosis. Due to DNA incorporation, 5′-Azacytidine and its derivate 5′-Aza-2′-Deoxycytidine have considerable cytotoxicity, in addition to their unspecific de-methylating activity. Hydralazine is not incorporated into DNA and recruits crucial steps in dynamic DNA de-methylation involving Tet3-mediated formation of 5-hydroxymethyl-cytosine (5hmC), 5-formyl-cytosine (5fC), and 5-carboxyl-cytosine (5caC) with subsequent Tdg-mediated excision and replacement with naked cytosine (Fig. 12A–C). Due to the CXXC motif target selectivity of Tet proteins, which directs Tet3 directly to the CpG islands within the *RASAL1* promoter, one can speculate that the de-methylating activity is more specific for aberrantly methylated genes as compared to 5′-Azacytidine, possibly accounting to its benign toxicity (Bechtel et al., 2010, Xu et al., 2012). Due to the prominent functional contribution of *RASAL1* promoter CpG island methylation to progression of chronic kidney disease, tools to specifically de-methylate *RASAL1* should be developed and explored in the future.

Our study adds further evidence for the utility of *RASAL1* methylation as a biomarker of renal fibrosis, which is not surprising as several transcriptional profiling studies revealed that RASAL1 expression is consistently decreased in kidney biopsies from patients with chronic kidney disease (Tampe et al., 2014). Importantly, we demonstrate for the first time that circulating methylated *RASAL1* CpG island promoter fragments correlate with degree of intrarenal *RASAL1* methylation and degree of fibrosis, similar to increased levels of methylated CpG fragments which can be detected in cancer patients. While the mechanism of how methylated promoter CpG island fragments are released into the circulation is not clear, we suspect that they are unspecifically liberated from injured cells, similar to what is observed in solid tumors. Because levels of methylated *Rasal1* CpG island promoter fragments decreased in mice which were treated with de-methylating and anti-fibrotic agent 5′-Azacytidine or Hydralazine, we speculate that circulating methylated DNA fragment levels dynamically reflect *RASAL1* CpG island promoter methylation within the kidney and may have utility to monitor effectiveness of de-methylating and anti-fibrotic therapies. As such decrease in circulating methylated *Rasal1* CpG island promoter fragments in mice correlated with blunted kidney fibrosis upon de-methylating therapy, it is attractive to speculate that decreased levels of methylated *RASAL1* DNA fragments and blood pressure-independent attenuation of CKD progression which were observed in patients which had received low-dose Dihydralazine are also reflective of its potential reno-protection.

Our finding that Hydralazine inhibits renal fibrosis due to the induction of active DNA de-methylation begs the question of why such a beneficial effect has gone unnoticed for a drug that has been used for more than 50 years. In this regard, existing data on a potential reno-protective effect of Hydralazine are inconclusive. While Hydralazine was long part of the standard-triple anti-hypertensive regimen (Hydrochlorothiazide, Propranolol, and Hydralazine), it was largely replaced when cardio-protective efficacy of ACEIs and ARBs was proven in large clinical trials (Heagerty et al., 1982). Hydralazine/Dihydralazine today is mainly used as third-line drug in patients with therapy-resistant hypertension (Leonetti et al., 1990). One reason the reno-protective effects of Hydralazine/Dihydralazine have never been documented may well be, that to our knowledge, such clinical trials to assess reno-protective effects have never been conducted. The rapid improvement of renal excretory function upon Hydralazine/Dihydralazine medication was observed soon after its introduction and attributed to unspecified vascular and hemodynamic effects at that time (Hollander et al., 1956, Pierpont et al., 1980). Because Hydralazine/Dihydralazine treatment is typically initiated at low doses of 12.5 mg (to avoid reflex tachycardia) before doses are increased to typical anti-hypertensive regimen of 100 mg, it is based on our clinical analysis tempting to speculate that those dynamics of excretory kidney function are a direct reflection of Hydralazine/Dihydralazine used. Few small clinical studies report delay of progression of CKD upon medication with Hydralazine/Dihydralazine (Mogensen, 1976, Parving et al., 1993). Studies which analyzed reno-protective efficacy of Hydralazine in rodent models of chronic kidney disease revealed conflicting results (Soto et al., 2004, Velasquez et al., 1997, Djukanovic et al., 1994). It is tempting to speculate that Hydralazine dosage might play a role in conflicting studies. It is well established that Hydralazine exerts its de-methylating activity at lower doses than its anti-hypertensive effect and that de-methylating efficacy does not increase at higher doses, as our studies showed (Zambrano et al., 2005). Our observation that high doses of Hydralazine induce adverse side effects such as Hif1α accumulation offers a possible explanation for conflicting studies (which were mostly performed at doses of 50 mg/kg/day and higher). Regarding the interpretation of the effect of Hydralazine/Dihydralazine therapy in existing patient cohorts, it has to be considered that doses used in patients are generally ten-fold lower than doses used in mice (due to resistance of mice to respond to anti-hypertensive action of Hydralazine).

Nevertheless, due to the high prevalence of hypertension in patients with CKD, low-dose Hydralazine/Dihydralazine as an add-on to current treatment regimens appears attractive as well as safe and might deserve reconsideration and prospective clinical testing. In combination with assessment of levels of methylated *RASAL1* DNA fragments in the blood without further need for biopsy, the possibility exists that this could be conducted in a biomarker therapy-stratified and biomarker monitored fashion.

## Author Contribution

BT performed and designed experiments, analyzed data and edited the manuscript. DT and WBW performed experiments and analyzed data. EMZ advised and edited the manuscript. GAM and MK provided human samples. RK edited the manuscript. MZ designed, performed and supervised experiments, analyzed data and wrote the manuscript.

## Fundings

This work was supported by the Else-Kröner-Fresenius Stiftung (P58/05 and P2014_11) and funds from the 10.13039/501100001659Deutsche Forschungsgemeinschaft (DFG-ZE523/4-1) to MZ. MZ and RK are also supported by NHI grant DK 081576. EZ is supported by 10.13039/501100001659DFG grant SFB1002/TPC01. BT and DT were supported by the “seed funding research program” of the Faculty of Medicine, Georg August University Göttingen (1402720 to BT and 1402910 to DT). WBW is supported by EU Marie Curie Career Integration Grant 293568, P7-PEOPLE-2011 and Margarete von Wrangell program of Baden-Württemberg. The funding sources had no involvement in the design, collection, analysis, interpretation, writing or decision to submit the article.

## Conflicts of Interest

The authors declare no conflicts of interest.
