# Supplementary material for: Induction of Tet3-dependent Epigenetic Remodeling by Low-dose Hydralazine Attenuates Progression of Chronic Kidney Disease
Source: eBioMedicine. 2014 Nov 8;2(1):19–36. doi: 10.1016/j.ebiom.2014.11.005 (PMC4337426; doi:10.1016/j.ebiom.2014.11.005)
Supplement: Supplementary file 1 — Supplementary material. [file mmc1.docx]

**Induction of Tet3-dependent epigenetic remodeling by low-dose Hydralazine attenuates progression of chronic kidney disease**

**Björn Tampe, MD^1#,2†^, Desiree Tampe, MD^1#,2†^, Elisabeth M. Zeisberg, MD^2,3#,4#^, Gerhard A. Müller, MD^1^, Wibke Bechtel-Walz, MD^2,5#^, Michael Koziolek, MD^1^, Raghu Kalluri, MD, PhD^2,6#^ and Michael Zeisberg, MD^1#,2*^**

**- Supplementary Material -**

*^1^ Department of Nephrology and Rheumatology, Göttingen University Medical Center, Georg August University, Robert Koch Street 40, Göttingen, Germany*

*^2^ Division of Matrix Biology, Department of Medicine, Beth Israel Deaconess Medical Center and Harvard Medical School, 330 Brookline Ave, Boston, Massachusetts, USA*

*^3^ Department of Cardiology and Pneumology, Göttingen University Medical Center, Georg August University, Robert Koch Street 40, Göttingen, Germany*

*^4^ German Center for Cardiovascular Research (DZHK), Robert Koch Street 40, Göttingen, Germany*

*^5^ Renal Division, University Hospital Freiburg, Hugstetter Street 55, Freiburg, Germany*

*^6^ Department of Cancer Biology and the Metastasis Research Center, University of Texas MD Anderson Cancer Center, 1515 Holcombe Blvd, Houston, Texas, USA*

*^#^ current institution*

*^†^* *equal contribution*

** corresponding author:*

Michael Zeisberg, MD

Professor for Experimental Nephrology

Department of Nephrology and Rheumatology

Göttingen University Medical Center

Georg August University

Robert Koch Str. 40

37075 Göttingen

Germany

Telephone: +49 551 3920076

Email: [mzeisberg@med.uni-goettingen.de](mailto:mzeisberg@med.uni-goettingen.de)

**
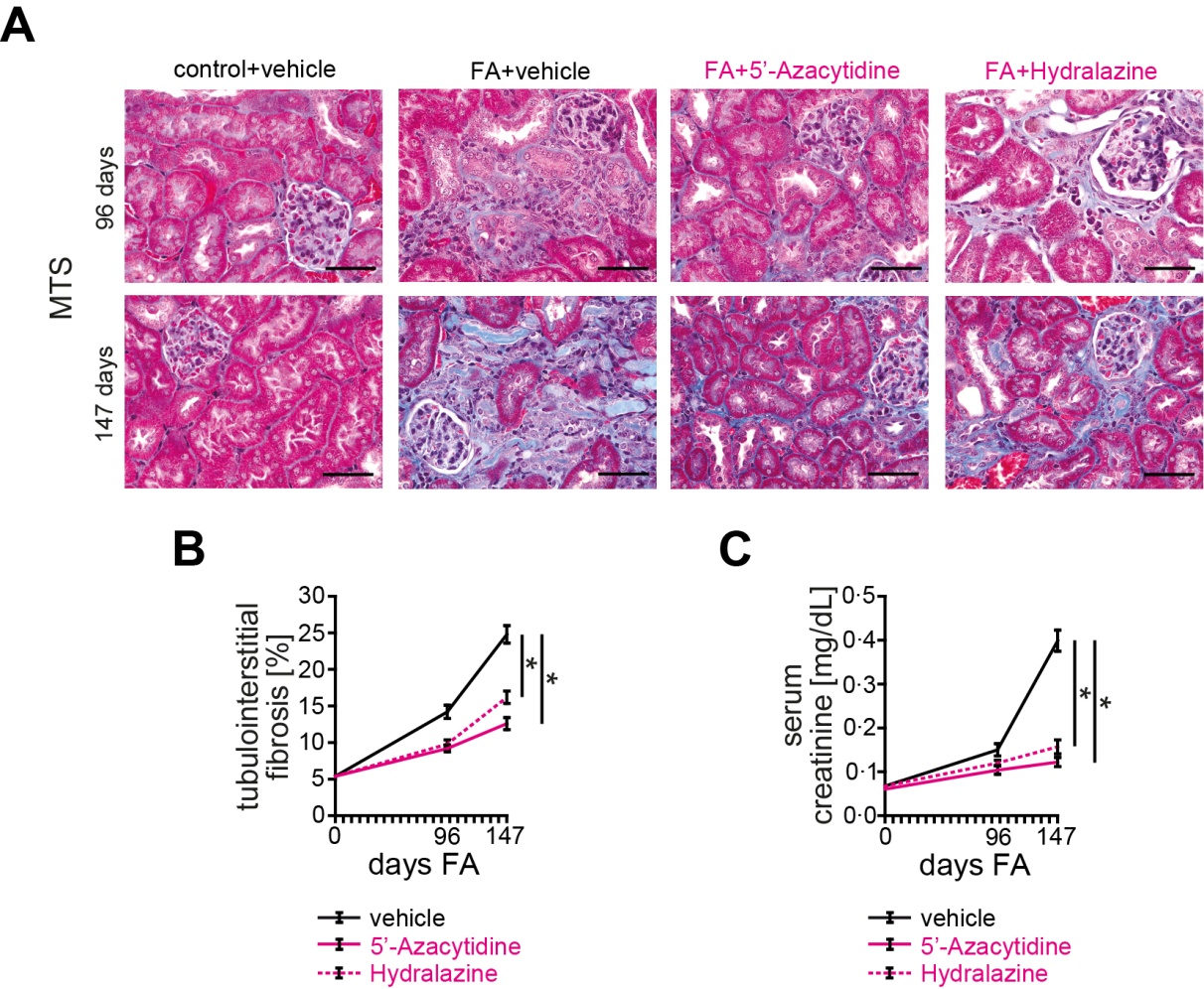
**

**Supplementary Figure 1. Effect of de-methylating 5´-Azacytidine or Hydralazine treatment on progression of experimental kidney fibrosis of folic acid-induced nephropathy (FA)**. (**A**) Representative photomicrographs of Masson's trichrome–stained (MTS) kidney sections from control mice (control+vehicle), mice that received folic acid and vehicle buffer PBS (FA+vehicle), 10mg/kg/day 5′-Azacytidine (FA+5’-Azacytidine), or 5mg/kg/day Hydralazine (FA+Hydralazine). Mice were sacrified 96 and 147 days after injection (original magnification x40, scale bars: 50 µm). (**B**) As compared to vehicle-treated fibrotic mice, interstitial fibrogenesis is ameliorated after treatment with de-methylating 5’-Azacytidine and Hydralazine. The graph summarizes average values at the indicated time points of each group (n=6 in each group, data are presented as means±s.e.m. **p<0.05*, values of *p* were calculated respective to vehicle-treated fibrotic mice). (**C**) Average serum creatinine concentrations at the indicated time points. As compared to fibrotic mice that received vehicle buffer PBS, ameliorated fibrosis correlated with blunted rise of creatinine levels in mice which had received 5’-Azacytidine or Hydralazine (n=6 in each group, data are presented as means±s.e.m. **p<0.05*, values of *p* were calculated respective to vehicle-treated fibrotic mice).

**
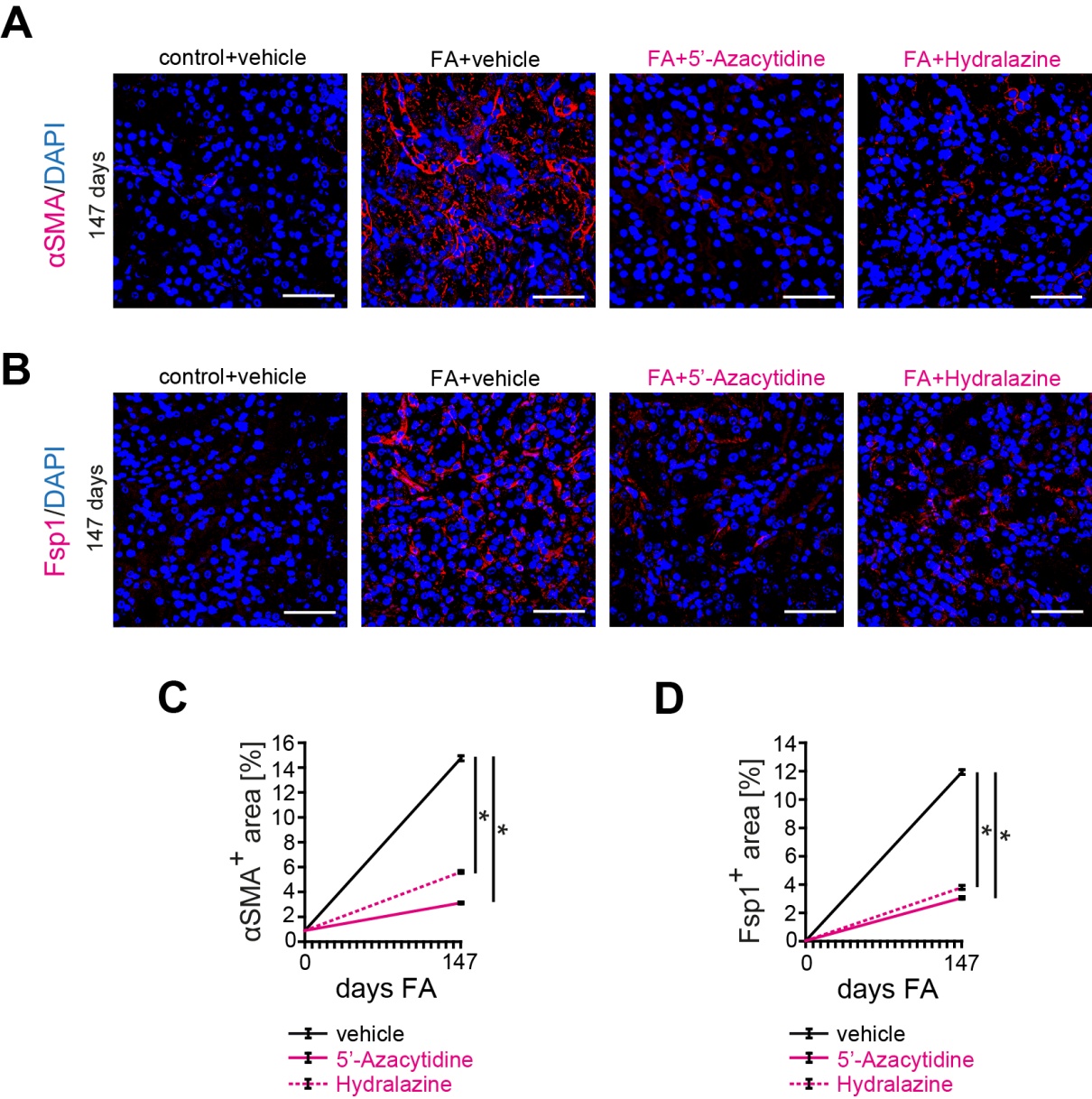
**

**Supplementary Figure 2. Hydralazine attenuates fibroblast accumulation in experimental renal fibrogenesis.** (**A-D**) Kidney sections were immunolabeled using primary antibodies against α-smooth muscle actin (αSMA) and fibroblast-specific protein 1 (Fsp1), representative confocal pictures are shown. As compared to vehicle-treated fibrotic mice (FA+vehicle), administration of 5’-Azacytidine (FA+5’-Azacytidine) and Hydralazine (FA+Hydralazine) ameliorated αSMA and Fsp1 accumulation (original magnification x63, scale bars: 50µm, n=8 in each group, data are presented as means±s.e.m. **p<0.05*, values of *p* were calculated respective to vehicle-treated FA mice).

**
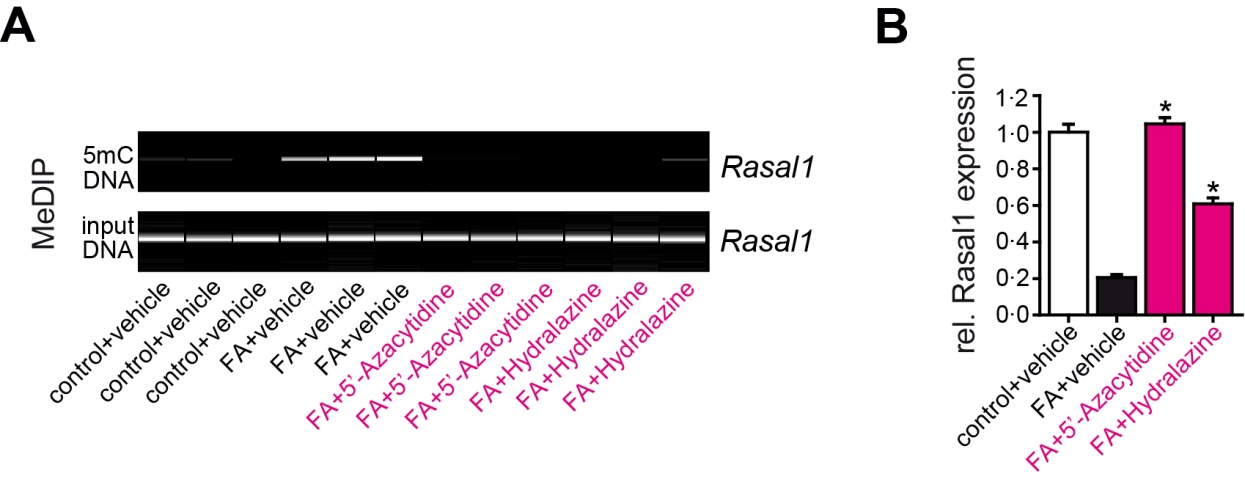
**

**Supplementary Figure 3. *Rasal1* promoter de-methylation upon treatment with 5’-Azacytidine and Hydralazine is associated with transcriptional Rasal1 induction.** (**A**) We performed methylated DNA immunoprecipitation (MeDIP) to assess the effect of anti-fibrotic 5’-Azacytidine and Hydralazine treatment on *Rasal1* methylation in mouse kidneys that were challenged with folic acid (FA). The upper picture displays a virtual gel image of *Rasal1* PCR products of captured (methylated, 5mC) DNA, the bottom picture displays *Rasal1* PCR products of input DNA (to control for equal loading in immunoprecipitation). *Rasal1* was methylated 147 days after folic acid induction of kidney fibrosis (FA+vehicle), whereas ameliorated fibrosis after treatment with de-methylating 5’-Azacytidine and Hydralazine (10mg/kg/day and 5mg/kg/day respectively) was associated with normalization of *Rasal1* promoter methylation. (**B**) Rasal1 mRNA expression was analyzed by qRT-PCR in RNA isolated from total kidneys. For statistical analysis, FA mice treated with either vehicle buffer PBS, 5’-Azacytidine or Hydralazine were compared to controls arbitrarily set to one. Rasal1 expression was suppressed in kidneys of mice which had been challenged with FA and which had received vehicle buffer PBS, treatment with de-methylating 5’-Azacytidine and Hydralazine restored suppressed Rasal1 mRNA expression (n=4 in each group, experiments were done in triplicate, data are presented as means±s.e.m. **p<0.05*, values of *p* were calculated respective to vehicle-treated FA mice).

**
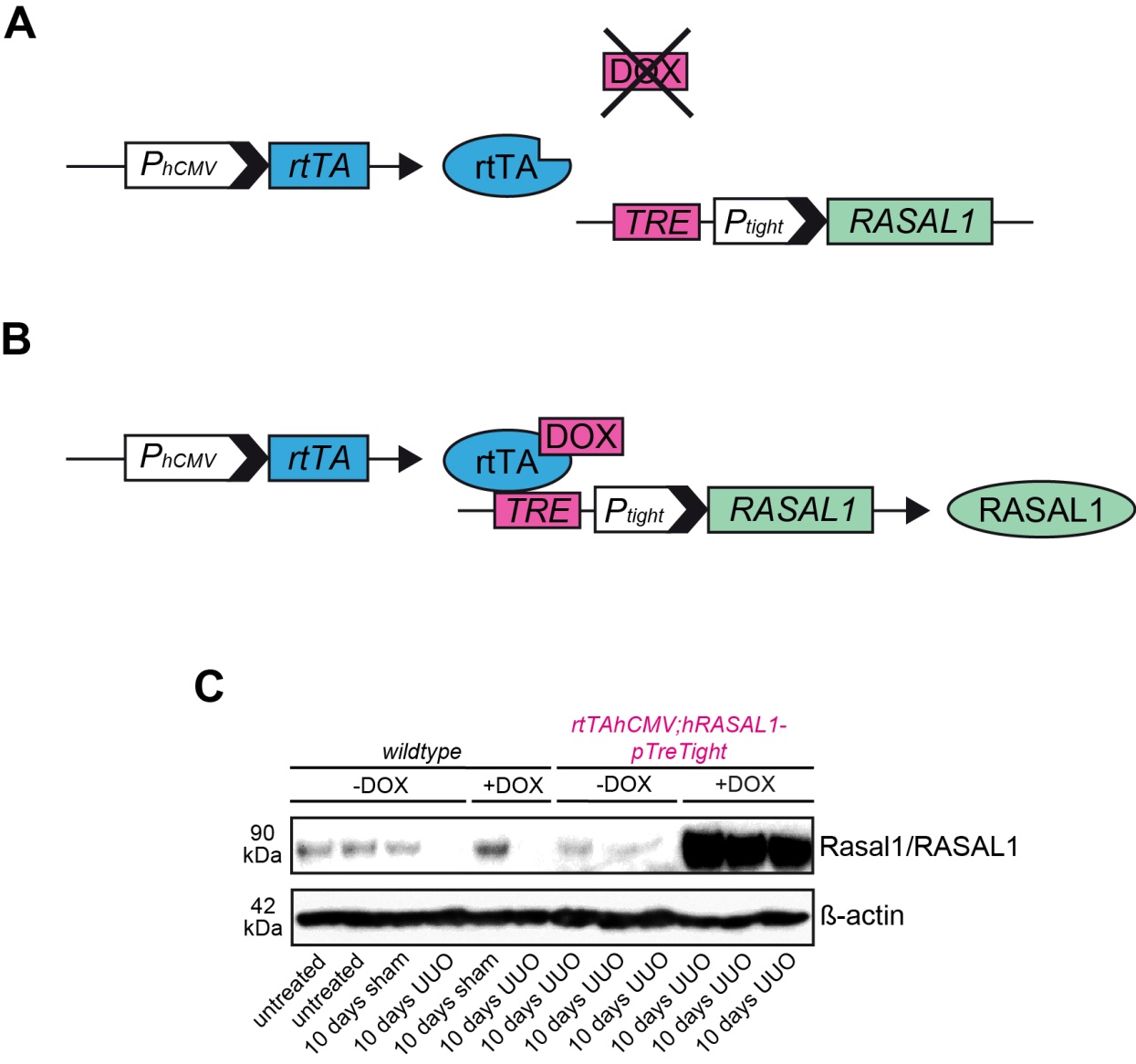
**

**Supplementary Figure 4. Generation of transgenic mice with an inducible *RASAL1* over-expression.** (**A,B**) Schematic illustration of *rtTAhCMV;hRASAL1-pTreTight* transgenic mice. In the absence of doxycycline (DOX), reverse tetracycline-controlled transactivator (rtTA) is not bound to the tetracycline-responsive element (TRE) and transgene-driven RASAL1 expression is absent. Treatment with doxycycline (DOX) results in complex formation with rtTA and binding to TRE with consecutive transgenic RASAL1 over-expression. (**C**) Protein levels of Rasal1/RASAL1 were analyzed by Western blot using a primary antibody targeting both, murine and human homologues. As compared to untreated and sham-operated controls, decreased Rasal1 expression observed in *wildtype* and non-induced *rtTAhCMV;hRASAL1-pTreTight* mice (*rtTAhCMV;hRASAL1-pTreTight*-DOX) challenged with unilateral ureteral obstruction (UUO) is restored by enforced expression of transgenic RASAL1 after induction (*rtTAhCMV;hRASAL1-pTreTight*+DOX).

**
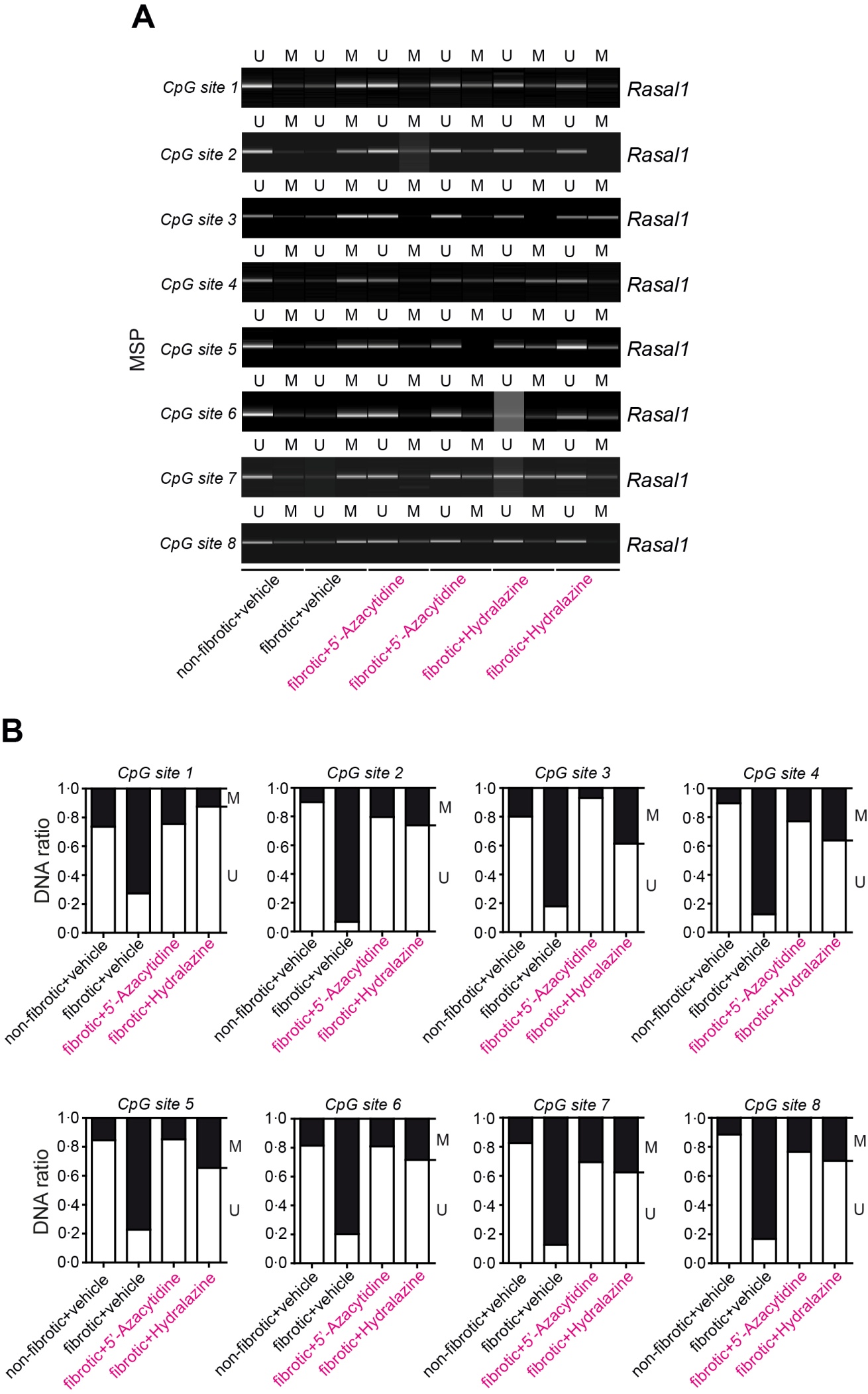
**

**Supplementary Figure 5. Treatment with de-methylating 5’-Azacytidine or Hydralazine mediates de-methylation of *Rasal1 CpG sites*.** (**A**) Methylation status of single *Rasal1* *CpG sites* within the *Rasal1* promoter was analyzed by methylation-specific PCR amplification (MSP) in primary mouse kidney fibroblasts out of non-fibrotic and fibrotic tissues after treatment with de-methylating 5´-Azacytidine (150µg/ml) or Hydralazine (50µg/ml) for 48 hours. Primers were targeting either unmethylated (U) or methylated (M) CpG dinucleotides in 8 different *Rasal1 CpG sites*, electrophoresis of PCR products was performed on a Bioanalyzer. Treatment normalized aberrant *Rasal1* *CpG* hypermethylation observed in fibrotic fibroblasts. (**B**) Band densities were analyzed using a Bioanalyzer, ratios for either unmethylated (U) or methylated (M) *Rasal1 CpG sites* are shown in response to vehicle buffer PBS, 5’-Azacytidine (150µg/ml) or Hydralazine (50µg/ml) for 48 hours.

**
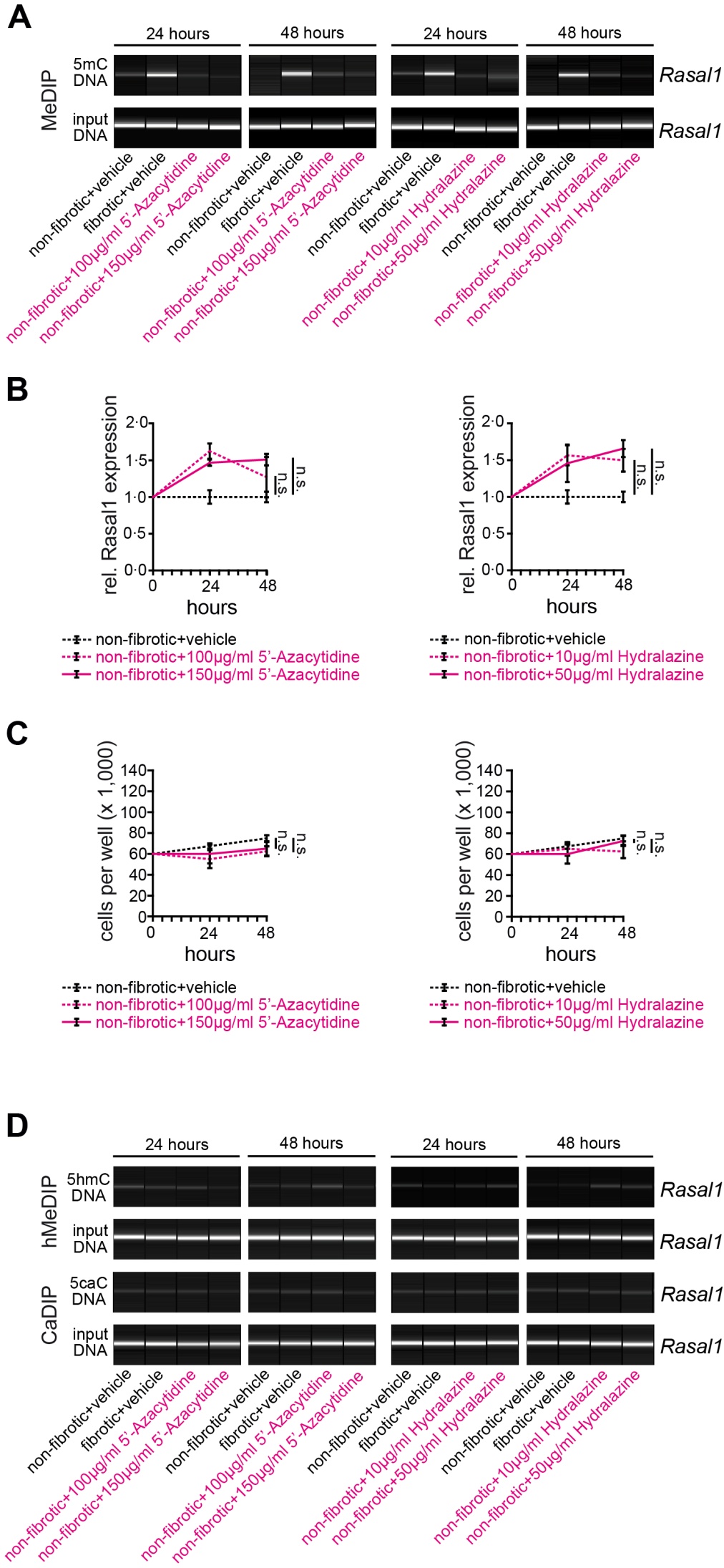
**

**Supplementary Figure 6. Treatment of fibroblast cultures from non-fibrotic kidneys with de-methylating 5’-Azacytidine or Hydralazine.** (**A-D**) De-methylating 5’-Azacytidine or Hydralazine had no effect on non-fibrotic fibroblast cultures. Non-fibrotic kidney fibroblasts were exposed to either vehicle buffer PBS, 5’-Azacytidine (100µg/ml or 150µg/ml), or Hydralazine (10µg/ml or 50µg/ml). Treatment had no effect on *Rasal1* promoter methylation, Rasal1 mRNA expression levels, intrinsic proliverative acticity or formation of *Rasal1* promoter hydroxymethylation or carboxylation (experiments were done in triplicate, data are presented as means±s.e.m. n.s. no significance, values of *p* were calculated respective to two days vehicle-treated non-fibrotic fibroblast cultures).

**
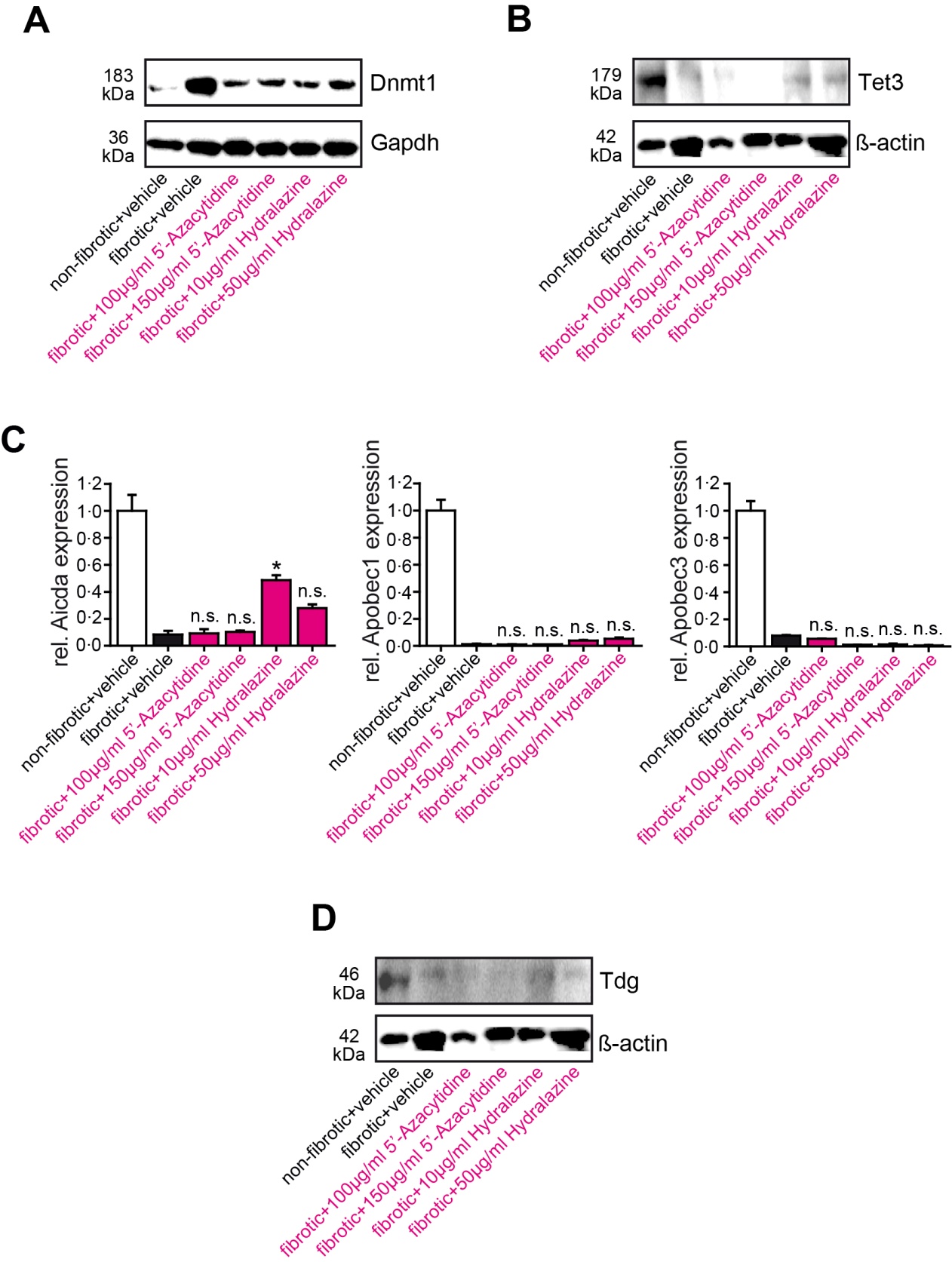
**

**Supplementary Figure 7. Impact of 5´-Azacytidine and Hydralazine on expression levels of genes facilitating active CpG methylation and hydroxymethylation.** (**A**) The upper picture shows Dnmt1 expression analyzed by Western blot, the bottom pictures display Gapdh as controls for equal loading. 5’-Azacytidine and Hydralazine treatment of primary fibrotic fibroblast cultures was associated with normalization of Dnmt1 expression levels. (**B**) As analyzed by Western blotting, Hydralazine treatment is associated with induction of DNA hydroxylase Tet3 in primary fibrotic fibroblast cultures. (**C**) Hydralazine treatment had no effect on mRNA expression levels of cytosine deaminases Aicda, Apobec1, or Apobec3 (experiments were done in triplicate, data are presented as means±s.e.m. **p<0.05*, n.s. no significance, values of *p* were calculated respective to vehicle-treated fibrotic cultures). (**D**) DNA glycosylase Tdg was induced after Hydralazine administration as analyzed by Western blot, β-actin was used as control for equal loading.

**
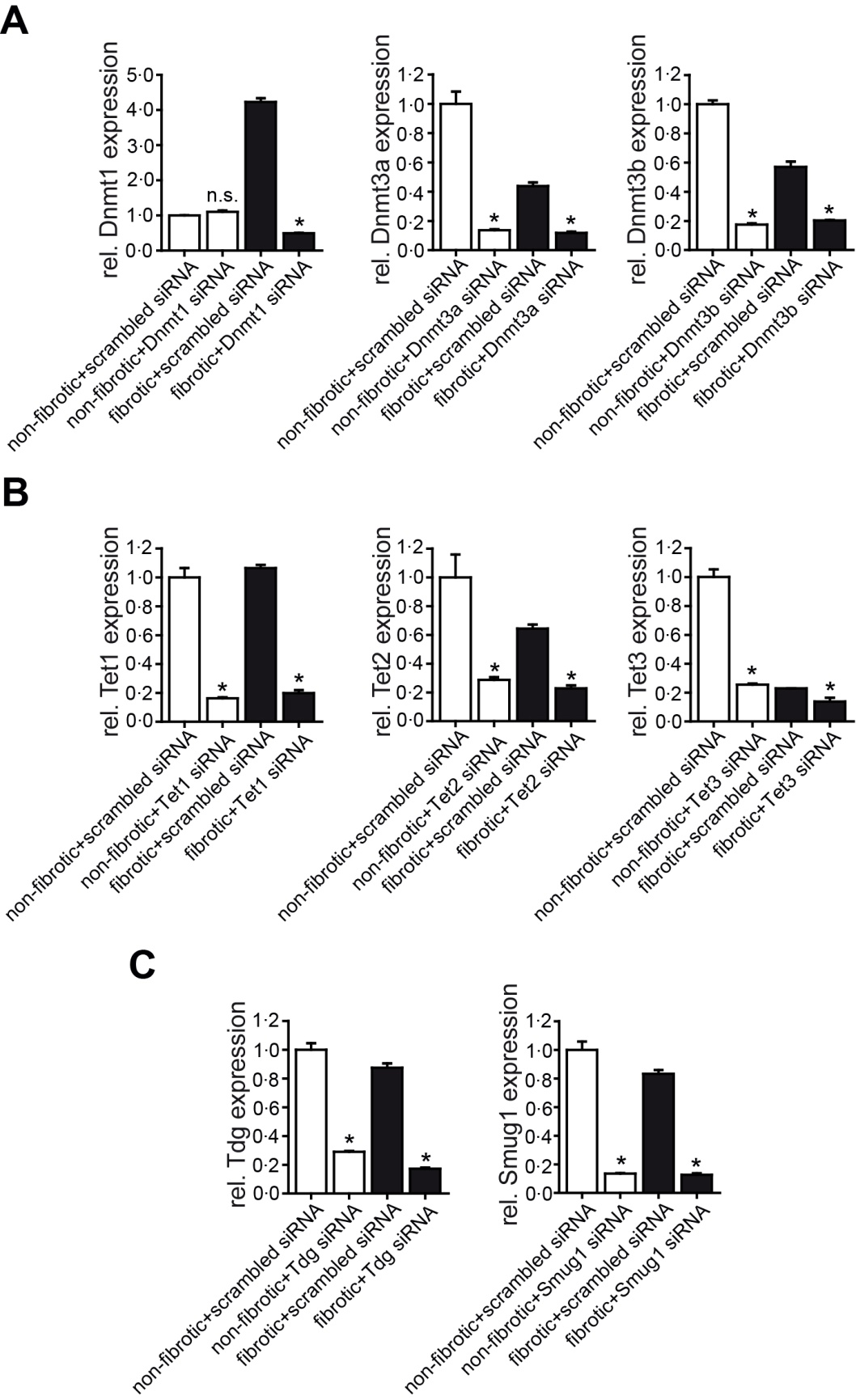
**

**Supplementary Figure 8. Knockdown efficacy of genes involved in active DNA methylation/de-methylation.** (**A-C**) SiRNA-mediated depletion of Dnmts, Tets, Aicda, Apobecs, Tdg and Smug1. Relative mRNA levels of genes involved in DNA methylation/de-methylation was analyzed by qRT-PCR after transfection of primary kidney fibroblasts with scrambled siRNA or siRNA specifically targeting Dnmts (Dnmt1, -3a, and 3b), Tets (Tet1, -2, and -3), Aicda, Apobecs (Apobec1 and -3), Tdg, and Smug1 (experiments were done in triplicate, data are presented as means. **p<0.05*, n.s. no significance, values of *p* were calculated respective to fibroblast cultures transfected with scrambled siRNA).

**
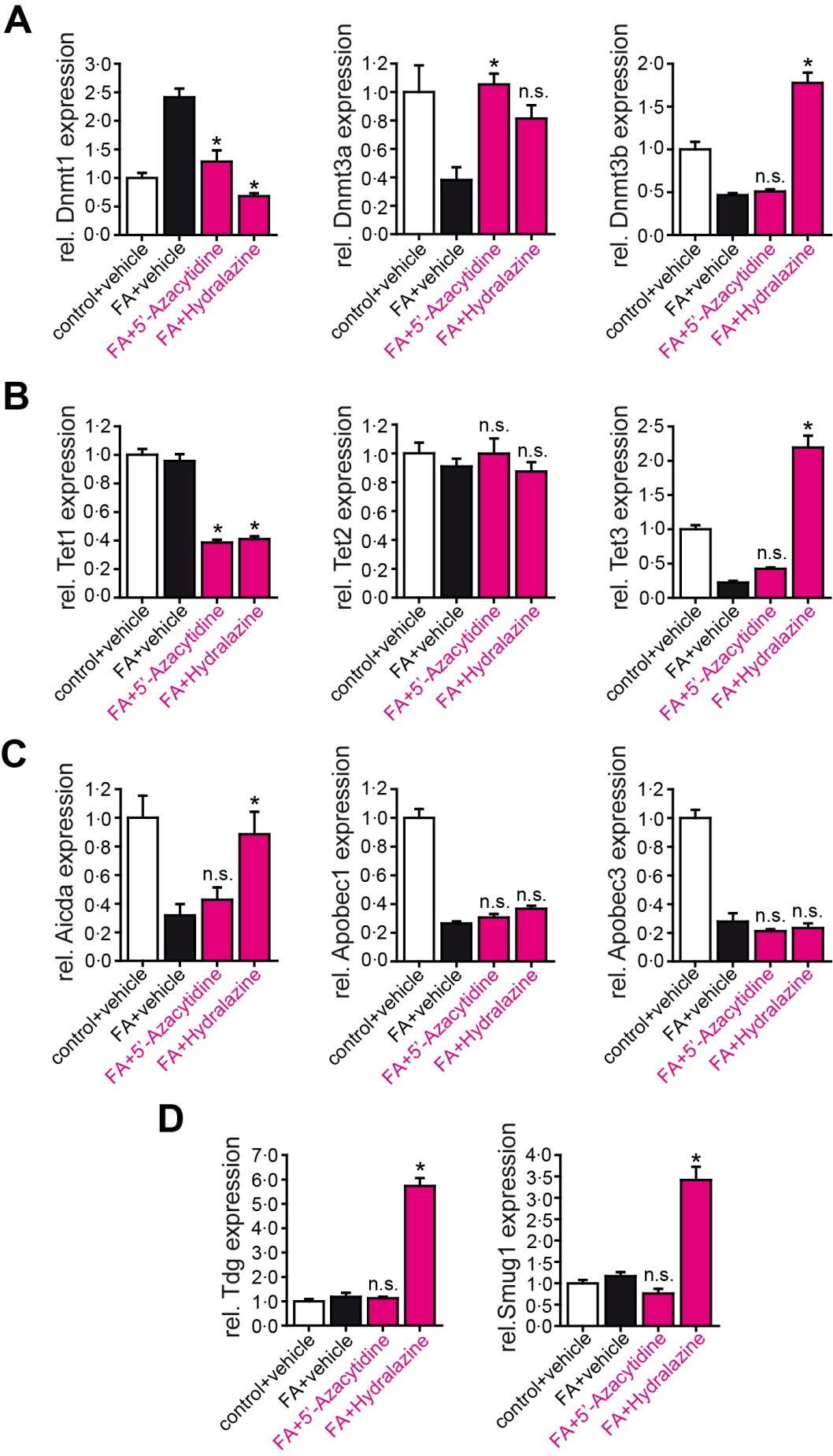
**

**Supplementary Figure 9. Impact of 5´-Azacytidine and Hydralazine on mRNA expression levels of genes involved in active CpG methylation and hydroxymethylation.** (**A**) Analyzed by qRT-PCR, mRNA expression levels of Dnmt1 and Dnmt3a were normalized after treatment with 5’-Azacytidine and Hydralazine. (**B**) Among Tet hydroxylases, Hydralazine induced expression of Tet3, but not of Tet1 or Tet2, whereas 5’-Azacytidine did not increase expression of either Tet gene. (**C,D**) Hydralazine treatment had no effect on expression levels of cytosine deaminases Aicda, Apobec1, or Apobec3, whereas DNA glycosylases Tdg and Smug 1 were induced after Hydralazine administration, 5’-Azacytidine did not impact mRNA expression levels (experiments were done in triplicate, data are presented as means±s.e.m. **p<0.05*, n.s. no significance, values of *p* were calculated respective to vehicle-treated fibrotic mice).


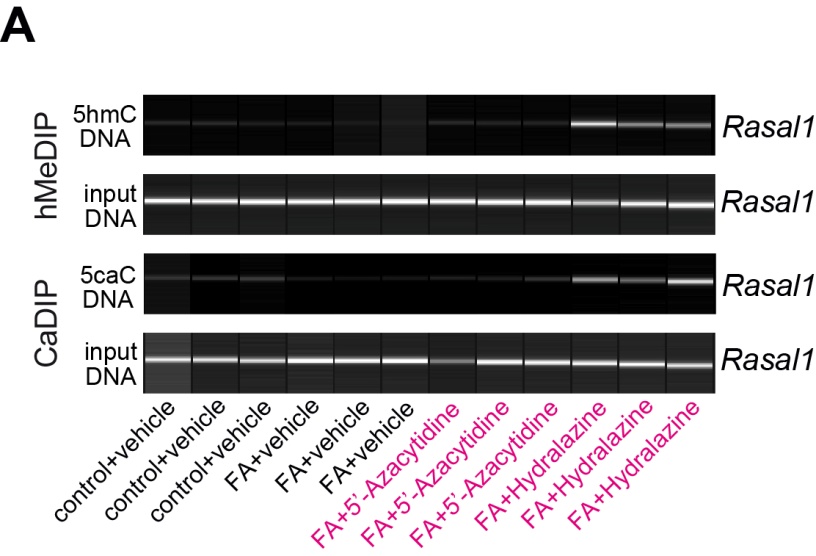


**Supplementary Figure 10. Normalization of *Rasal1* promoter methylation upon Hydralazine involves enzymatic de-methylation depend on cytosine hydroxymethylation and carboxylation.** (**A**) We performed DNA immunoprecipitations on hydroxymethylated (hMeDIP) and carboxylated cytosines (CaDIP) to assess the effect of 5’-Azacytidine and Hydralazine on enzymatic cytosine modifications involved in active DNA de-methylation in mice challenged with folic acid (FA). Whereas none of these modifications were observed in fibrotic mice treated with either vehicle buffer PBS (FA+vehicle) or 5’-Azacytidine (FA+5’-Azacytidine), treatment with Hydralazine (FA+Hydralazine) was associated with *Rasal1* hydroxymethylation and *Rasal1* carboxylation suggesting that de-methylating Hydralazine involves enzymatic modifications in normalization of *Rasal1* promoter methylation.


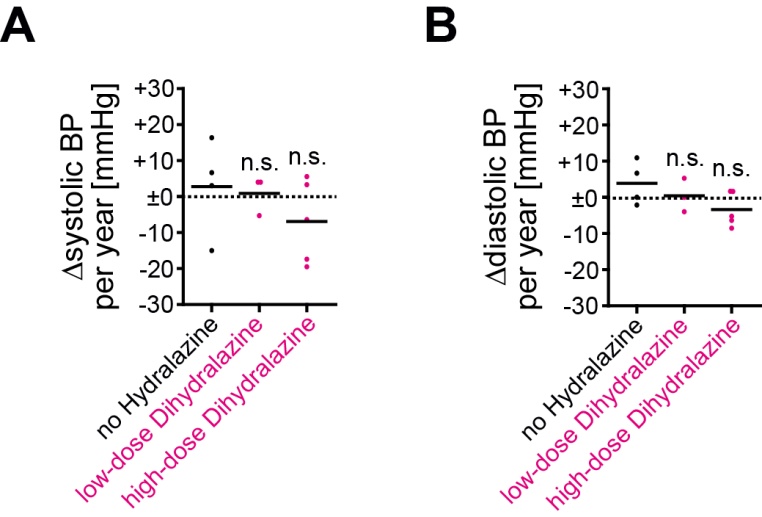


**Supplementary Figure 11. De-methylating Dihydralazine mediates reno-protection independent of systemic blood pressure effects.** (**A,B**) In hypertensive patients with CKD without Dihydralazine treatment (n=4), low-dose (n=3) or high-dose Dihydralazine medication (n=5) does not alter systolic or diastolic blood pressure over time (data are presented as scatter dot blots with lines at means. n.s. no significance, values of *p* were calculated respective to patients with no Dihydralazine medication).

| **target gene** | **primer forward sequence** |
| --- | --- |
| **supplier** | **primer reverse sequence** |
| mAicta | GCAGATGAGGACTACGAAGAAAT |
| Primerdesign, Southampton, UK | TGATAAGACTGTGAGTAAACTAATGAC |
| mApobec1 | CCAGAGACAGAGCAAGATGAGT |
| Primerdesign, Southampton, UK | CGGGGGTCAAAGAAGACTTCA |
| mApobec3 | CCCACCCAACCCCAAGTC |
| Primerdesign, Southampton, UK | GTCTTCAAAACAAAGCAAAACAACA |
| mRasal1 | CATCAGTGGCTTCCTCTTTCT |
| Primerdesign, Southampton, UK | GTCTGCGTGCTGGTCTCT |
| hRASAL1 | CGTGCTGGATGAGGACACTG |
| Eurofins MWG Operon | TCCCTGCTCAGCGAGATCTT |
| mSmug1 | GGAGAATGACCAATGTTACGAGAA |
| Primerdesign, Southampton, UK | ATGAAGTGTAGGAAGAGAGGAGAG |
| mTdg | CCGAAGACGCTCCTGTCC |
| Primerdesign, Southampton, UK | CTGGTTCCTGGGGCTCTG |
| mTet1 | TGAAGATGACAAGCAGCAAACC |
| Primerdesign, Southampton, UK | TTGTTGAGCGGAAGGTGTGT |
| mTet2 | GACTCAACGGTTATCAGGCTTTT |
| Primerdesign, Southampton, UK | CATTGCTCTTTATTCTTCCTCTGTAA |
| mTet3 | CTCCCCTGCTGTCTTCAGA |
| Primerdesign, Southampton, UK | CCTGAGGCTCTGTGGAAGTA |
| mGapdh | undisclosed |
| Primerdesign, Southampton, UK | undisclosed |
| hGAPDH | undisclosed |
| Primerdesign, Southampton, UK | undisclosed |

**Supplementary Table A. Oligonucleotide sequences for qRT-PCR analysis.**

| **primer** | **forward primer sequence** |
| --- | --- |
| **supplier** | **reverse primer sequence** |
| mRasal1 | CTGGCTCAGCCTCCTGTTCTG |
| Eurofins MWG Operon | CAGACAACCCCGATCCAGGACC |
| mRasal1-quantification | GCCGAGGGCTCAAAACTGAG |
| Eurofins MWG Operon | GCGGAGGCTCCCACGTCACCGGC |
| hRASAL1 | GCCCGCCCAGCCTGCTTGTCTGG |
| Eurofins MWG Operon | GGCAGGCAGCGCGCGGCCCTCCACC |
| hRASAL1-quantification | GCCAACTCACCAGGAGCCAGCGGC |
| Eurofins MWG Operon | CTACCGGCACCCCAGTCATGCGC |

**Supplementary Table B. Oligonucleotide sequences for SYBR-based amplification of methylated/hydroxymethylated/carboxylated *Rasal1*/*RASAL1*.**

| **primer** | **forward primer sequence** |
| --- | --- |
| **supplier** | **reverse primer sequence** |
| CpG site 1-U | TTATGAAAGGTTTAAGTTGTG |
| Eurofins MWG Operon | GAATTTTTTAAGAATTTGGGTG |
| CpG site 1-M | TTATGAAAGGTTTAAGTCGTG |
| Eurofins MWG Operon | GAATTTTTTAAGAATTTGGGTG |
| CpG site 2-U | GAATTTGGTTAAGTGTTTGAG |
| Eurofins MWG Operon | GAATTTTTTAAGAATTTGGGTG |
| CpG site 2-M | GAATTTGGTTAAGTGTTCGAG |
| Eurofins MWG Operon | GAATTTTTTAAGAATTTGGGTG |
| CpG site 3-U | TTGTAGATGGTTTTTATTGTG |
| Eurofins MWG Operon | GAATTTTTTAAGAATTTGGGTG |
| CpG site 3-M | TTGTAGATGGTTTTTATCGTG |
| Eurofins MWG Operon | GAATTTTTTAAGAATTTGGGTG |
| CpG site 4-U | TGTTGTTTGGGTGGTGTTATT |
| Eurofins MWG Operon | GAATTTTTTAAGAATTTGGGTG |
| CpG site 4-M | TGTCGTTCGGGCGGTGTTATT |
| Eurofins MWG Operon | GAATTTTTTAAGAATTTGGGTG |
| CpG site 5-U | TGGTAGTTGGTGATGTGGGAG |
| Eurofins MWG Operon | GAATTTTTTAAGAATTTGGGTG |
| CpG site 5-M | TGGTAGTCGGTGACGTGGGAG |
| Eurofins MWG Operon | GAATTTTTTAAGAATTTGGGTG |
| CpG site 6-U | GGGAGTTTTTGTTGGTATATT |
| Eurofins MWG Operon | GAATTTTTTAAGAATTTGGGTG |
| CpG site 6-M | GGGAGTTTTCGTTGGTATATT |
| Eurofins MWG Operon | GAATTTTTTAAGAATTTGGGTG |
| CpG site 7-U | ATTAGATTTTTAGGGAGTGTG |
| Eurofins MWG Operon | GAATTTTTTAAGAATTTGGGTG |
| CpG site 7-M | ATTAGATTTTTAGGGAGCGTG |
| Eurofins MWG Operon | GAATTTTTTAAGAATTTGGGTG |
| CpG site 8-U | TGTGTTGTTTGTTGTTTAGAG |
| Eurofins MWG Operon | GAATTTTTTAAGAATTTGGGTG |
| CpG site 8-M | TGCGTCGTTTGTCGTTTAGAG |
| Eurofins MWG Operon | GAATTTTTTAAGAATTTGGGTG |

**Supplementary Table C. Oligonucleotide sequences for SYBR-based amplification of unmethylated/methylated *Rasal1 CpG sites*.**

| **biopsy ID** | **histology** | **fibrosis**  **[%]** | **eGFR**  **[mL/min]** | **creatinine**  **[mg/dL]** | **BUN**  **[mg/dL]** |
| --- | --- | --- | --- | --- | --- |
| 3570 | C3 glomerulonephritis | 0 | 99 | 1.0 | 8 |
| 3724 | Fabry disease | 10 | 82 | 0.8 | 12 |
| 3468 | chronic transplant rejection | 25 | 28 | 2.2 | 32 |
| 3582 | FSGS | 30 | 52 | 1.2 | 21 |
| 3538 | nephrosclerosis | 35 | 13 | 5.0 | 79 |
| 3732 | FSGS | 35 | 31 | 1.6 | 22 |
| 3616 | transplant nephrosclerosis | 40 | 17 | 2.9 | 57 |
| 3522 | FSGS | 50 | 16 | 3.6 | 52 |
| 3562 | mesangioproliferative GN | 60 | 27 | 2.7 | 28 |

**Supplementary Table D. Corresponding patient data of human kidney biopsies. Biopsy ID.** Tissues were named in order of the date of biopsy. **Histology.** Summary of the pathological diagnoses of the biopsies. **Fibrosis.** The biopsies were scored by a pathologist for fibrosis (relative extend of tubulointerstitial fibrosis is shown in %). **eGFR.** Estimated glomerular filtration rate [mL/min] was calculated using MDRD equation. **Creatinine/BUN.** Serum creatinine [mg/dL] and blood urea nitrogen [mg/dL] was measured at time of biopsy.

| **group** | **arterial**  **HTN** | **CKD** | **Dihydralazine**  **medication** | | **gender w:m** | **age**  **[years]** | **eGFR**  **[mL/min]** | **creatinine**  **[mg/dL]** | **BUN**  **[mg/mdL]** |
| --- | --- | --- | --- | --- | --- | --- | --- | --- | --- |
| 1 | yes | no | no | | 1:3 | 68±8 | 64±8 | 1*.*0±0*·*1 | 20±2 |
| 2 | yes | no | yes | | 1:3 | 63±4 | 69±6 | 0*.*9±0*·*1 | 18±1 |
|  | | | | ***p* value <0*.*05** | | **no** | **no** | **no** | **no** |
| 3 | yes | yes | no | | 3:1 | 72±3 | 27±3 | 2*.*0±0*·*2 | 30±6 |
| 4 | yes | yes | yes | | 2:2 | 72±4 | 25±1 | 2*.*1±0*·*1 | 44±9 |
|  | | | | ***p* value <0*.*05** | | **no** | **no** | **no** | **no** |

**Supplementary Table E. Corresponding patient data of human blood samples.** Patients were grouped for arterial hypertension (HTN), chronic kidney disease (CKD) and Dihydralazine medication. **Gender w:m.** Number of female and male patients in each group. **Age.** Average age±s.e.m. in each group at time of analysis. **eGFR.** Estimated glomerular filtration rate [mL/min] was calculated using MDRD equation, data is presented as means±s.e.m. **Creatinine and BUN.** Serum creatinine [mg/dL] and blood urea nitrogen [mg/dL] were measured at time of analysis, data is presented as means±s.e.m. ***P* value <0*.*05.** Values of *p* were calculated for each parameter comparing group 1 and 2 or group 3 and 4, respectively.

| **patient** | **disease** | **eGFR**  **[mL/min]** | **creatinine**  **[mg/dL]** | **BUN**  **[mg/dL]** |
| --- | --- | --- | --- | --- |
| control patient | C3 glomerulonephritis | 99 | 1*.*0 | 8 |
| CKD patient 1 | FSGS | 26 | 1*.*9 | 37 |
| CKD patient 2 | nephrosclerosis | 27 | 2*.*3 | 76 |

**Supplementary Table F. Clinical data of CKD patients newly put on Dihydralazine medication.** Summary of the pathological diagnoses of the patients. **eGFR.** Estimated glomerular filtration rate [mL/min] was calculated using MDRD equation. **Creatinine/BUN.** Serum creatinine [mg/dL] and blood urea nitrogen [mg/dL] was measured at time of biopsy.

| **group** | **Dihydralazine**  **medication** | **gender w:m** | **age**  **[years]** | | **follow-up**  **[months]** | **baseline creatinine**  **[mg/dL]** | **baseline BP s/d**  **[mmHg]** |
| --- | --- | --- | --- | --- | --- | --- | --- |
| 1 | no | 1:3 | 44±8 | | 34±2 | 1*.*8±0*·*5 | 120±8/74±2 |
| 2 | low-dose | 2:1 | 58±18 | | 26±6 | 1*.*8±0*·*6 | 135±3/72±6 |
|  | | | | ***p* value <0.05** | | **no** | **no** |
| 3 | high-dose | 4:1 | 67±7 | | 27±5 | 1*.*9±0*·*5 | 143±9/76±6 |
|  | | | | ***p* value <0*.*05** | | **no** | **no** |

**Supplementary Table G. Clinical data of patients on long-term Dihydralazine medication due to arterial hypertension.** Patients were grouped for no, low-dose or high-dose Dihydralazine medication. **Gender w:m.** Number of female and male patients in each group. **Age.** Average age±s.e.m. in each group at time of first visit. **Follow-up.** Average months of follow-up±s.e.m. in each group. **Baseline creatinine.** Baseline serum creatinine [mg/dL] was measured at time of first visit, data is presented as means±s.e.m. **Baseline BP s/d.** Baseline blood pressure [mmHg] was measured at time of first visit, data is presented as means±s.e.m. of systolic/diastolic measurements. ***P* value <0*.*05.** Values of *p* were calculated for each parameter comparing Dihydralazine-treated groups 2 or 3 to group 1.
